# Transcriptional analysis of immune response genes during pathogenesis of cytomegalovirus retinitis in mice with murine acquired immunodeficiency syndrome

**DOI:** 10.1371/journal.ppat.1009032

**Published:** 2020-11-06

**Authors:** Jessica J. Carter, Jesse M. Gardner, Brent P. Poling, Madeline M. Welch, Judee Grace E. Nemeno, John E. Houghton, Richard D. Dix

**Affiliations:** 1 Department of Biology, Viral Immunology Center, Georgia State University, Atlanta, Georgia, United States of America; 2 Department of Ophthalmology, Emory University School of Medicine, Atlanta, Georgia, United States of America; Oklahoma State Univeristy, UNITED STATES

## Abstract

Human cytomegalovirus (HCMV) is an opportunistic human herpesvirus that causes a sight-threatening retinitis in immunosuppressed patients, especially those with AIDS. Using an established model of experimental murine cytomegalovirus (MCMV) retinitis in mice with retrovirus-induced immunodeficiency (MAIDS), we have been attempting to define with greater clarity the immunologic mechanisms that contribute to the progression of AIDS-related HCMV retinitis in the unique immunosuppressive setting of HIV infection. Toward this end, we provide herein a comprehensive assessment of immune response gene expression during the onset and development of MAIDS-related MCMV retinitis employing NanoString nCounter. In so doing, we analyzed and compared the intraocular expressions of 561 immune response genes within MCMV-infected eyes of groups of healthy mice, MCMV-infected mice with MAIDS of 4 weeks’ (MAIDS-4) duration, and MCMV-infected eyes of mice with MAIDS of 10 weeks’ (MAIDS-10) duration. These animal groups show a progression of retinal disease from absolute resistance to retinitis development in healthy mice to the development of classic full-thickness retinal necrosis in MAIDS-10 mice but through an intermediate stage of retinal disease development in MAIDS-4 mice. Our findings showed that increased susceptibility to MCMV retinitis during the progression of MAIDS is associated with robust upregulation or downregulation of a surprisingly large number of immune response genes that operate within several immune response pathways often unique to each animal group. Analysis of 14 additional immune response genes associated with programmed cell death pathways suggested involvement of necroptosis and pyroptosis during MAIDS-related MCMV retinitis pathogenesis. Use of the NanoString nCounter technology provided new and unexpected information on the immunopathogenesis of retinitis within MCMV-infected eyes of mice with retrovirus-induced immunosuppression. Our findings may provide new insights into the immunologic events that operate during the pathogenesis of AIDS-related HCMV retinitis.

## Author summary

The immunodeficiency caused by HIV during the progression of AIDS creates a unique systemic immunosuppressive environment that often leads to the development of opportunistic virus infections. Among these is a sight-threatening retinitis caused by human cytomegalovirus (HCMV). While the clinical and histopathologic features of AIDS-related HCMV retinitis have been well documented, the immunologic mechanisms that continue to operate during HIV-induced immunosuppression and contribute to the onset and development of retinal tissue destruction caused by ocular HCMV infection in patients with AIDS remain unexplored. Herein, we report the first comprehensive analysis of a total of 571 immune response genes that are expressed during the pathogenesis of experimental murine cytomegalovirus (MCMV) retinitis in mice at different stages of retrovirus-induced immunosuppression (MAIDS) who exhibit different susceptibilities to retinal disease development. Our finding that different patterns of immune response gene expression are observed at different stages of retrovirus immunosuppression during increased susceptibility to retinal necrosis development adds new knowledge to our understanding of the contributions of unique innate and adaptive immune pathways towards the pathogenesis of AIDS-related HCMV retinitis and may extend to other AIDS-related opportunistic virus infections.

## Introduction

The unique immunosuppressive environment created by HIV infection during the development of AIDS resulted in the emergence of a significant number of diseases caused by opportunistic viruses that prior to AIDS were no more than rare medical curiosities. One such AIDS-related opportunistic disease that appeared as a consequence of HIV-induced immunosuppression is a sight-threatening retinitis caused by human cytomegalovirus (HCMV), a β herpesvirus [1,2]. Although HCMV retinitis had been documented occasionally in a patient immunosuppressed for solid-organ or bone-marrow allografts [3], the appearance of an AIDS-related HCMV retinitis was quickly observed in up to 42% of this patient population at the outset of AIDS in the United States [4]. Today the number of cases of AIDS-related HCMV retinitis in the United States has decreased significantly due to the use of combination antiretroviral therapy (ART) but nonetheless remains an ophthalmologic problem in some parts of the world in patients who do not have access to ART or who do not respond to ART [5–8].

The clinical and histopathologic features of AIDS-related HCMV retinitis have been well documented [9–12]. To extend this knowledge to include the virologic, immunologic, and pathogenic mechanisms that operate to allow the onset and progression of HCMV retinitis in patients with AIDS, we have been using an experimental animal model of murine cytomegalovirus (MCMV) retinitis that develops in mice with retrovirus-induced immunodeficiency (MAIDS) [13]. MAIDS-related MCMV retinitis mimics AIDS-related HCMV retinitis in many ways. These include the appearance of generalized lymphadenopathy, polyclonal B cell activation, hypergammaglobulinemia, and a Th1 to Th2 shift in cytokine profile accompanied by progressive dysfunction of cellular immunity that takes place over weeks following systemic infection with an immunosuppressive murine retrovirus mixture [14]. These events culminate in the development of MAIDS by 8 to 10 weeks after retrovirus infection that allows susceptibility to a retinitis in eyes inoculated with MCMV [15]. Importantly, MAIDS-related MCMV retinitis exhibits histopathologic features identical to those of AIDS-related HCMV retinitis. These include the emergence of a full-thickness retinal necrosis with prominent cytomegalic cells that develops within MCMV-infected retinal tissues at a frequency of 80 to 100% by 10 days after intraocular MCMV inoculation [13].

Previous work by us has used this animal model of MAIDS-related MCMV retinitis as an experimental platform to understand with greater clarity the pathogenesis of AIDS-related HCMV retinitis as dictated within the host by the unique immunologic milieu created by an immunosuppressive retrovirus. Past emphasis has been on various components of innate and adaptive immunity of the host with more recent focus being given to individual cytokines and a number of programmed cell death pathways [16]. While informative, these past studies have not been at the depth needed to identify and compare the extraordinary number of immune response genes expressed simultaneously within the ocular compartment at critical times during the evolution of MAIDS-related MCMV retinitis. In an attempt to overcome this obstacle, we employed NanoString nCounter technology which allows for the direct measurement of immune response gene expression levels without amplification during the onset and progression of retinitis within MCMV-infected eyes of mice with MAIDS. Herein, we report the intraocular expression of 575 immune response genes within MCMV-infected eyes of mice at different stages of MAIDS development and compared with MCMV-infected eyes of immunologically normal mice. As expected, our results show that intraocular MCMV infection of mice with MAIDS results in the upregulation or downregulation of immune response genes associated with several distinct immune response pathways during retinitis development. In addition, some immune response genes and pathways identified in this investigation have been surprising and not recognized by us previously, thereby extending our understanding of the role of various immune responses toward the pathogenesis of MAIDS-related MCMV retinitis. Furthermore, we have observed dramatic differences in the expression of immune response genes to intraocular virus infection depending on the resistance or degree of susceptibility of MCMV-infected eyes to retinitis development.

## Results

### Hierarchical clustering analysis of 561 immune response gene transcripts within MCMV-infected eyes of healthy mice, MAIDS-4 mice, and MAIDS-10 mice

Several prior investigations by us have confirmed major differences in the susceptibility to development of full-thickness retinal necrosis when comparing the MCMV-infected eyes of healthy mice with the MCMV-infected eyes of mice at different stages of retrovirus-induced immunosuppression [13,17]. Whereas the retinal architecture of MCMV-infected eyes of healthy mice remain normal without histopathologic evidence of retinal disease, the MCMV-infected eyes of mice with MAIDS of 10-weeks duration (MAIDS-10 mice) show a progressive development of retinitis at 3, 6, and 10 days after intraocular MCMV inoculation that culminates in severe, full-thickness retinal necrosis at 10 days after inoculation [13]. Intermediate between the two histopathologic extremes of the eyes of healthy mice and MAIDS-10 mice inoculated with virus are the MCMV-infected eyes of mice with MAIDS of 4-weeks duration (MAIDS-4 mice) that show only mild retinal pigment epithelium (RPE) proliferation with preservation of the neurosensory retina but with an absolute absence of full-thickness retinal necrosis throughout the course of infection [17]. These diverse pathogenic outcomes among healthy mice, MAIDS-4 mice, and MAIDS-10 mice following intraocular MCMV infection therefore allowed us the unique opportunity to compare MCMV-infected eyes of these animal groups for simultaneous detection and quantification of transcript expression for a large number of immunologic pathways and related components throughout the present investigation.

Initial studies were performed to determine the overall change in expression of 561 immune defense genes during the pathogenesis of MCMV retinitis. This was accomplished using a commercial NanoString nCounter Murine Immunology Panel that included 15 additional internal reference and housekeeping genes [18]. The left eyes of groups of healthy mice (n = 3), MAIDS-4 mice (n = 3), and MAIDS-10 mice (*n* = 3) were inoculated with MCMV; the contralateral right eyes of each animal group were mock-infected with maintenance medium only and served as internal controls. At 3, 6, and 10 days after intraocular inoculation, individual MCMV-infected and mock-infected eyes were collected from all animal groups, subjected to total RNA extraction, and the MCMV-infected or mock-infected eyes were pooled by groups and subjected to NanoString nCounter analysis. S1 Table details our findings for MCMV-infected eyes for each of the 561 immune defense gene analyzed whether upregulated or downregulated for each animal group of this investigation. Included are their overall fold-change expression profiles together with *p* values at 3, 6, and 10 days postinfection when compared with mock-infected eyes.

**Fig 1:**
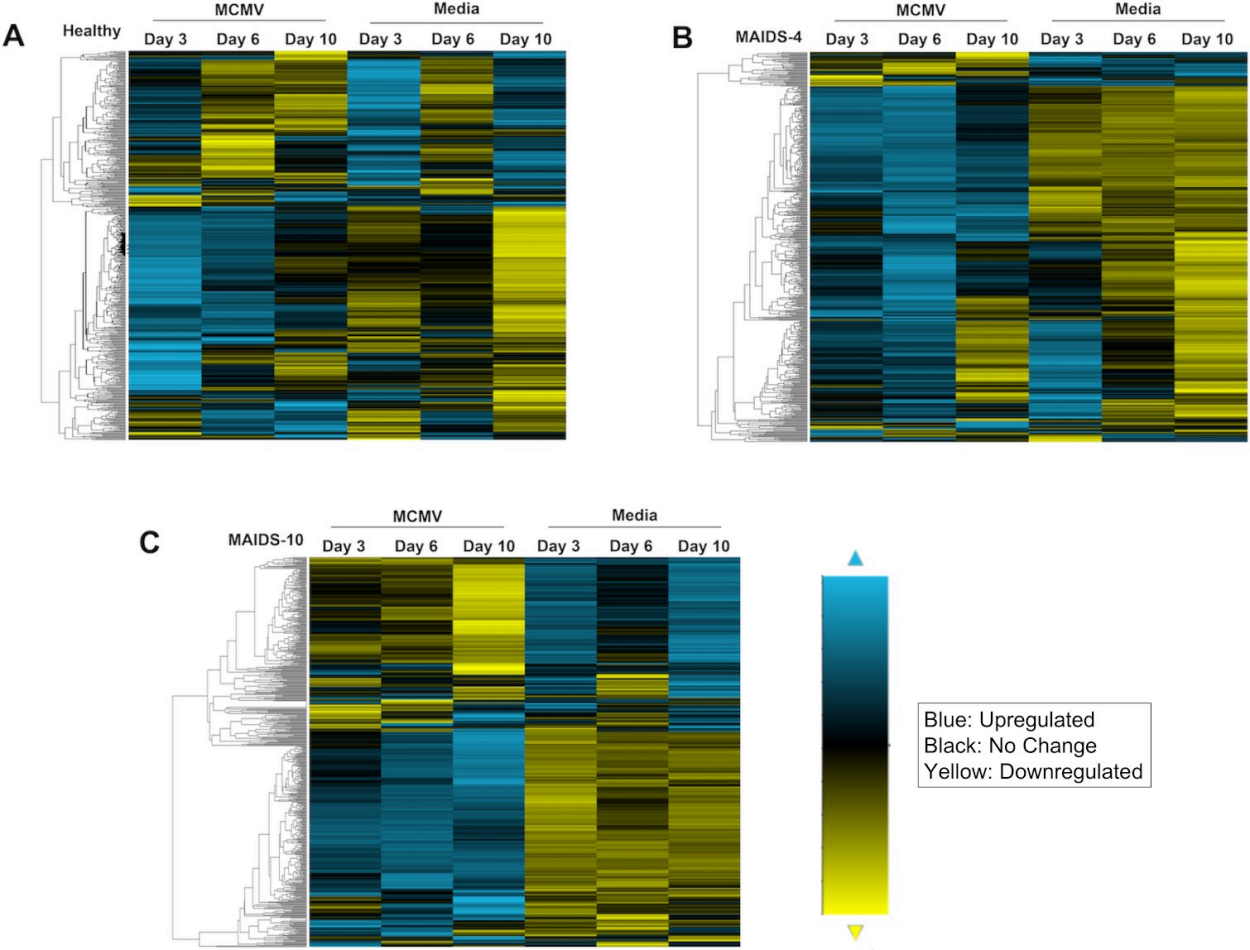
Hierarchical clustering analysis of 561 immune response gene transcripts within MCMV-infected eyes of healthy mice, MAIDS-4 mice, and MAIDS-10 mice. Whole MCMV-infected eyes (MCMV) and mock-infected (Media) eyes were collected 3, 6, and 10 days after intraocular inoculation from groups of (A) healthy mice (n = 3), (B) MAIDS-4 mice (n = 3), and (C) MAIDS-10 mice (n = 3). Total RNA was extracted from individual MCMV-infected eyes or mock-infected eyes and pooled for each group. 100ng of RNA from each group were loaded onto a Murine Immunology Panel, specifically designed for the NanoString nCounter. Hierarchical clustering analysis for each of the 561 genes was performed using the nSolver software. The bar indicates range of transcriptional activity with blue indicating upregulation, yellow indicating downregulation, and black indicating no change in mRNA expression.

Hierarchical clustering analysis of the expression of 561 immune response gene transcripts analyzed for each group of MCMV-infected eyes or mock-infected eyes are presented in Fig 1. Inspection of these data indicates that patterns of gene expression differed greatly when comparing mock-infected and MCMV-infected eyes at each time point examined. More importantly, the patterns of immune response gene expression were remarkably distinct for each animal group, reflecting resistance or the degree of susceptibility to MCMV retinitis development. The MCMV-infected eyes of healthy mice without MAIDS that show absolute resistance to retinitis development [17] nonetheless exhibited active gene expression (Fig 1A). At least some of this transcriptional activity might be attributed to an intraocular trauma created in response to the needlestick that takes place during mock infection. In comparison, MCMV-infected eyes of MAIDS-4 mice and MAIDS-10 mice also showed active gene expression but with increased upregulation of genes when compared with MCMV-infected eyes of healthy mice. Of interest, MCMV-infected eyes of MAIDS-4 mice that fail to develop full-thickness retinal necrosis but nonetheless exhibit RPE proliferation showed an unexpected and extensive upregulation of a majority of the 561 immune response genes investigated at day 6 after virus inoculation (Fig 1B) when compared with retinitis-susceptible MCMV-infected eyes of MAIDS-10 mice at day 6 after virus inoculation (Fig 1C). This outcome was particularly surprising because of our previous observation that the MCMV-infected eyes of both animal groups harbor high but equivalent amounts of infectious virus [17]. Moreover, distinctly different expression patterns for individual immune response genes were observed within the MCMV-infected eyes of MAIDS-10 mice following development of full-thickness retinal necrosis but not within MCMV-infected eyes of MAIDS-4 mice resistant to full-thickness retinal necrosis development.

### Comparison of MCMV-infected eyes of healthy mice, MAIDS-4 mice, and MAIDS-10 mice for the expression of genes associated with distinct immunologic pathways

**Fig 2:**
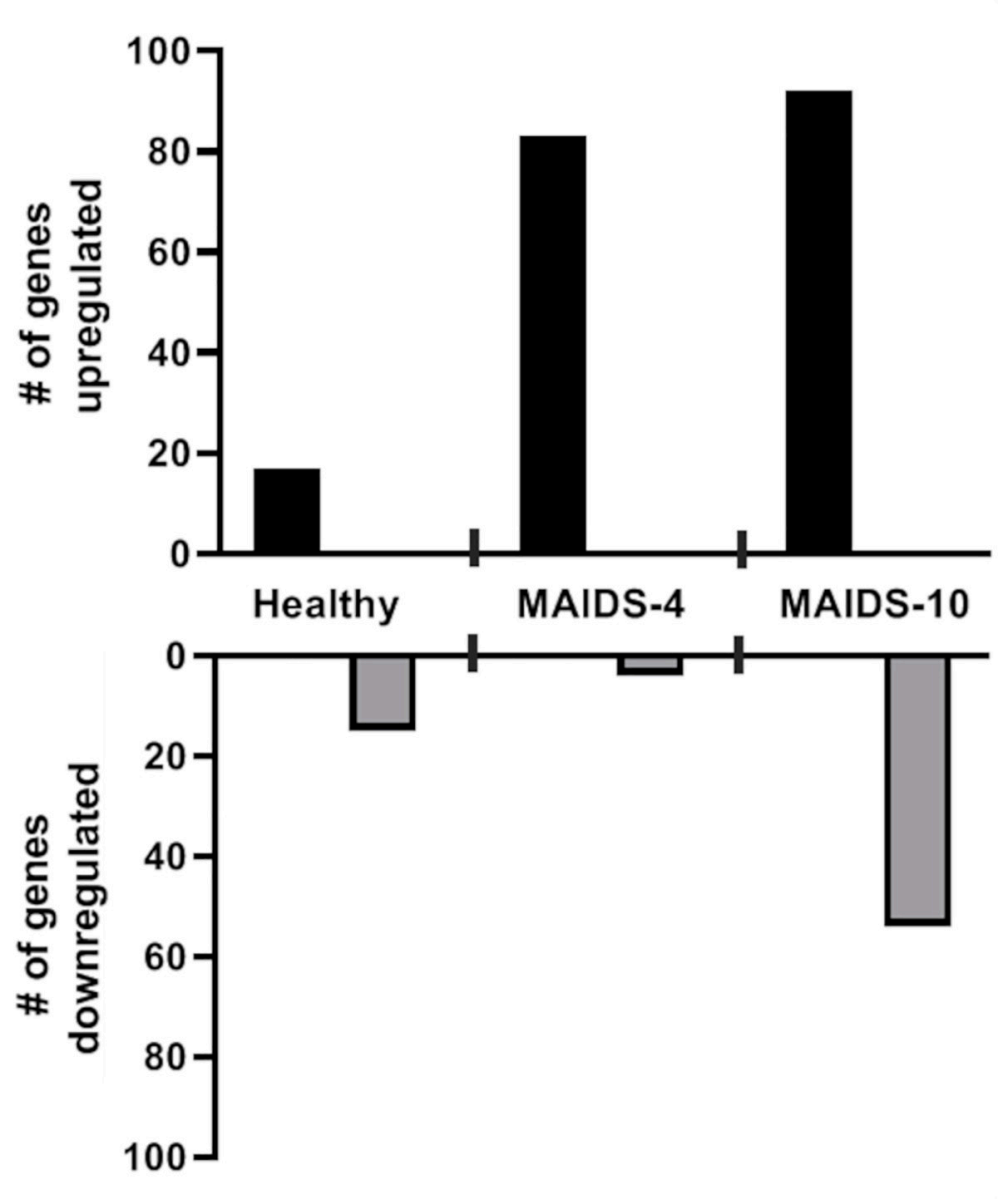
Comparison of the number of upregulated and downregulated immune-response genes for MCMV-infected eyes of groups of healthy mice, MAIDS-4 mice, and MAIDS-10 mice. Comparison of the number of differentially expressed immune-response genes with a fold change of two-fold or greater for MCMV-infected eyes collected from groups of healthy mice (n = 3), MAIDS-4 mice (n = 3), and MAIDS-10 mice (n = 3) when compared with mock-infected eyes at all time points examined.

We next processed the hierarchical clustering analysis of the 561 immune response gene transcripts from MCMV-infected eyes of healthy mice, MAIDS-4 mice, and MAIDS-10 mice at all time points examined for their involvement in 32 distinct immunologic pathways using the NanoString nSolver software. After determining the fold-change upregulation or downregulation of differentially expressed immune-response genes of MCMV-infected eyes when compared with mock-infected eyes for each animal group, a fold change of less than two was used to exclude that particular gene from further analysis. This approach revealed 17 genes were upregulated and 15 genes were downregulated within MCMV-infected eyes of healthy mice, 83 genes upregulated and 4 genes downregulated within MCMV-infected eyes of MAIDS-4 mice, and 92 genes upregulated and 54 genes downregulated within MCMV-infected eyes of MAIDS-10 mice (Fig 2). An increasing trend in immune response-related gene activity was observed when MCMV-infected eyes of healthy mice were compared to MCMV-infected eyes of MAIDS-4 mice and then compared to MCMV-infected eyes of MAIDS-10 mice. This trend is presumably due to an increased susceptibility of the MCMV-infected eyes of these animal groups toward development of full-thickness retinal necrosis as suggested in the resulting heatmaps (Fig 1).

**Fig 3:**
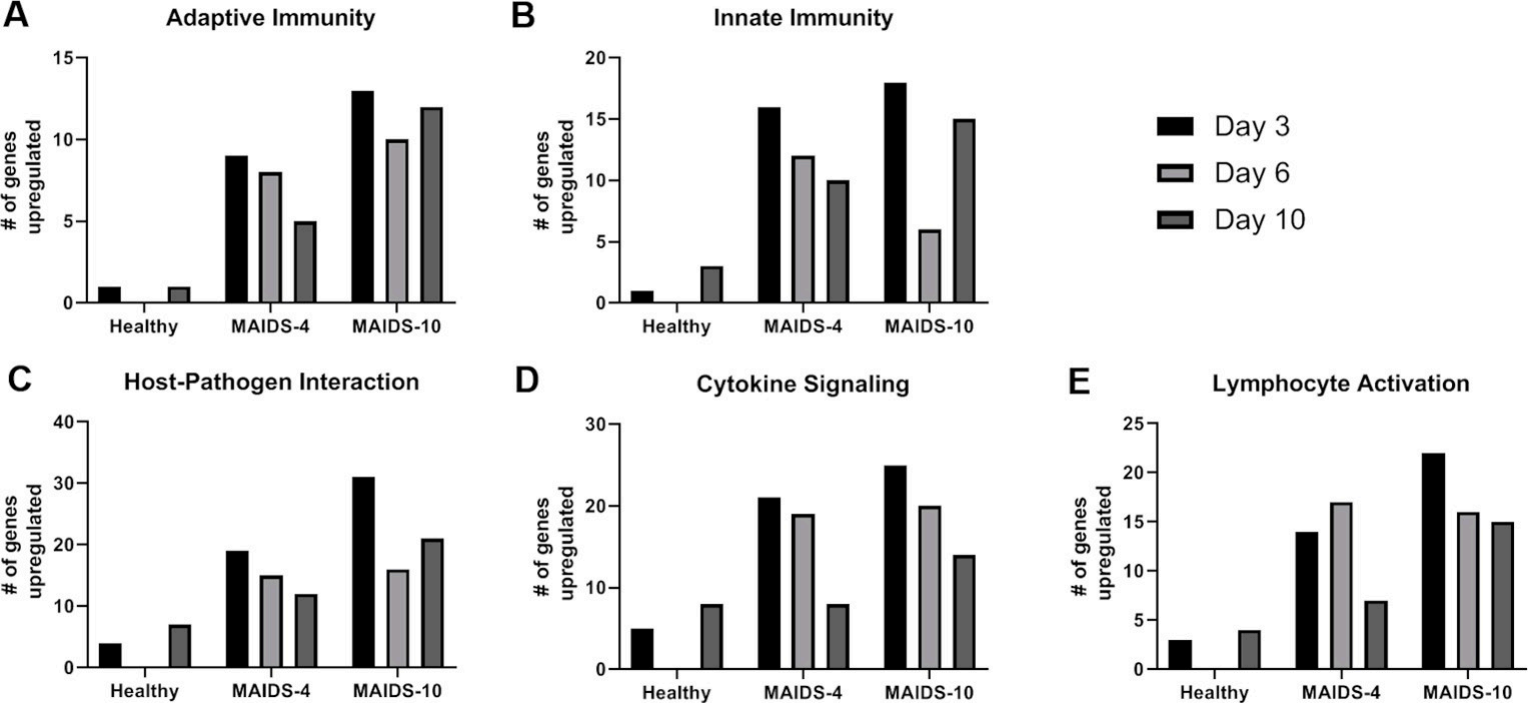
Number of upregulated immune response genes within MCMV-infected eyes in groups of healthy mice, MAIDS-4 mice, and MAIDS-10 mice when categorized according to major immunologic pathways. Transcriptional activity of immune response genes of MCMV-infected eyes in groups of healthy mice (n = 3), MAIDS-4 mice (n = 3), and MAIDS-10 mice (n = 3) that registered a fold change of > two, when compared with mock-infected eyes at 3, 6, or 10 days postinfection. These genes were categorized into five NanoString-defined immunologic pathways that exhibited the most robust upregulation. Immunologic pathways included the broad categories of: (A) Adaptive Immunity, (B) Innate Immunity, (C) Host-Pathogen Interaction, (D) Cytokine Signaling, and (E) Lymphocyte Activation.

This trend also continued when the differentially expressed immune response genes showing upregulation of activity were organized into NanoString-defined immunologic pathways for each animal group with the understanding that each gene could be involved in multiple pathways. Categorization into immunologic pathways revealed that five of the 32 NanoString-defined pathways exhibited relatively substantial stimulation of gene activity within MCMV-infected eyes when compared with other pathways of MCMV-infected eyes of healthy mice, MAIDS-4 mice, and MAIDS-10 mice during progressive susceptibility to MCMV retinitis. Those pathways showing the most robust stimulation in gene activity included pathways associated with the broad categories of adaptive immunity, innate immunity, host-pathogen interactions, cytokine signaling, and lymphocyte activation (Fig 3). More functionally focused pathways such as those involved with NF-κB signaling, toll-like receptor signaling, NOD-like receptor signaling, chemokine signaling, type 1 interferon signaling, type 2 interferon signaling, tumor necrosis factor (TNF) family signaling, MHC class I antigen presentation, and phagocytosis and degradation also showed less robust but nonetheless heightened gene activity when comparing MCMV-infected eyes of MAIDS-4 and MAIDS-10 mice with MCMV-infected eyes of healthy mice (S1 Fig). Overall, these findings demonstrate that gene activity associated with immunologic pathways becomes progressively and dramatically more active in number and function as MCMV-infected eyes become progressively more susceptible to the onset and development of full-thickness retinal necrosis as retrovirus-induced immunosuppression ensues. Moreover, this progressive gene activity is far more complex than was originally thought and appears to involve a large number of immune pathways of which several were never considered by us to be involved in the evolution of cytomegalovirus retinitis in retrovirus-immunosuppressed hosts.

### A comparison of the top 15 differentially expressed immunologic-associated genes within MCMV-infected eyes of healthy mice, MAIDS-4 mice, and MAIDS-10 mice

In an attempt to provide a more quantitative analysis of the progressive response(s) of the 561 immune response genes analyzed within MCMV-infected eyes as they relate to the progressive susceptibility to retinitis development, we focused on 15 differentially expressed genes which showed the greatest upregulation of gene expression at 3, 6, or 10 days after intraocular MCMV inoculation and compared these levels with those of mock-infected eyes for groups of healthy mice, MAIDS-4 mice, and MAIDS-10 mice. Table 1 summarizes our findings for MCMV-infected eyes for each animal group with respect to individual genes, their known function(s), their overall fold change at peak expression along with their *p* values when compared with mock-infected eyes [19–61]. In agreement with our previous observations, the fold-change for stimulation of the top 15 individual genes within MCMV-infected eyes appeared to increase markedly from healthy mice to MAIDS-4 mice and MAIDS-10 mice, seemingly as a reflection of increased susceptibility to the onset and development of MCMV retinitis among these animal groups, especially MAIDS-4 animals when compared with MAIDS-10 animals. While the average fold-increase for peak expression in activity of these 15 genes was 3.04 for MCMV-infected eyes of healthy mice, the average fold-increase for peak expression in activity increased to 20.14 and 20.74 for MCMV-infected eyes of MAIDS-4 mice and MAIDS-10 mice, respectively, suggesting a far more dynamic intraocular gene transcription activity in response to MCMV infection during progressive retrovirus-induced immunosuppression than during immunocompetence. It is also noteworthy that a subset of individual genes exhibited remarkable transcription activity such as *ccl5*, a gene encoding for a chemokine associated with inflammation [51], that showed an 83.32-fold increase in activity within MCMV-infected eyes of MAIDS-10 mice susceptible to full-thickness retinal necrosis development when compared to mock-infected eyes.

**Table 1:** Summary of the 15 differentially expressed immune response genes showing the greatest upregulation of activity within MCMV-infected eyes of groups of healthy mice, MAIDS-4 mice, and MAIDS-10.

Top 15 Genes Upregulated in MCMV infected Eyes of Healthy Mice			
Gene	Function [Ref.]	Peak Expression	p value
Bst1	Facilitates pre-B-cell growth and induces cell migration [19]	3.25	0.0298
Casp3	Activation plays a role in the execution -phase of apoptosis [20]	2.36	0.0117
Ccl3	Associated with macrophage recruitment [21]	2.28	0.0324
Ccl9	Induces chemotaxis of CD4+ T cells, CD8+ T cells, and monocytes [22]	3.09	0.0107
Cd2	Regulates natural killer cell lytic activity and proinflammatory cytokine production [23]	2.74	0.0365
Clec5a	Involved in neutrophil extracellular trap formation and proinflammatory cytokine production [24]	3.74	0.0244
Emr1	Murine marker of macrophages (F4/80) [25]	2.48	0.0280
H2-K1	Bind to and present antigens derived from pathogens onto the cell surfaces for T cell recognition [26]	4.37	0.0444
Ifnar2	Part of IFN-α and IFN-β receptor and critical for antiviral immunity [27]	2.10	0.0377
Itgb2	Involved in extravasation into tissues during infection or injury [28]	3.03	0.0016
Jak2	Involved in signal transduction of interferon and cytokine signaling [29]	2.70	0.0131
Ptafr	Involved in proinflammatory signaling [30]	2.53	0.0230
Ptgs2	Involved in the production of prostacyclin, expressed in inflammation [31]	3.80	0.0011
Stat2	Aids in the activation of the transcription of interferon stimulated genes [32]	4.37	0.0350
Tgfb1	Inhibits the actions of T cells and the secretion of IFN-γ, TNF-α, and interleukins [33, 34, 35]	2.75	0.0091
Top 15 Genes Upregulated in MCMV infected Eyes of MAIDS-4 Mice			
Gene	Function [Ref.]	Peak Expression	p value
Ccl12	Attracts eosinophils, monocytes, and lymphocytes to the site of infection [36]	10.88	0.0450
Ccl2	Involved in chemotaxis and regulating inflammation [37]	51.23	0.0353
Ccl7	Promotes the recruitment of monocytes and neutrophils to the site infection [38]	34.39	0.0350
Ccr5	Acts as a receptor for chemokines [39]	11.10	0.0123
Cfb	Regulates the alternative pathway of the complement system [40]	27.79	0.0030
Cxcl10	Attracts CD8+ and CD4+ T cells to the site of inflammation [41]	30.24	0.0374
Cxcl9	Attracts T cells to the site of inflammation [42]	28.12	0.0413
Icos	Involved in the induction and regulation of Th1, Th2, and Th17 immunity [43]	10.01	0.0354
Ifit2	Plays a role in the stimulation of interferons as part of an anti-viral response [44]	17.09	0.0008
Irgm1	Involved in the polarization of M1 [inflammatory driven] macrophages [45]	12.16	0.0450
Itgal	Involved in leukocyte cellular adhesion and costimulatory signaling [46]	11.48	0.0185
Lilrb3	Functions as an inhibitory receptor to help balance the function of innate immune cells [47]	9.59	0.0407
Lilrb4	Transduces a negative signal that inhibits stimulation of the immune response [48]	16.53	0.0209
Ptprc	Suppresses JAK kinases as a negative regulator of cytokine signaling [49]	10.54	0.0490
Slamf7	Induces B cell proliferation [50]	21.03	0.0411
Top 15 Genes Upregulated in MCMV infected Eyes of MAIDS-10 Mice			
Gene	Function [Ref.]	Peak Expression	p value
Ccl2	Involved in chemotaxis and regulating inflammation [37]	35.19	0.0323
Ccl5	Promotes the recruitment of leukocytes to the site of infection [51]	83.32	0.0263
Cd274	Plays a major role in suppressing the adaptive arm of the immune system [52]	7.36	0.0145
Ctss	Degrades antigenic proteins for antigen presentation [53]	12.69	0.0443
Cybb	Involved in the formation of reactive oxygen species [54]	9.83	0.0191
Fcgr3	Participates in signal transduction triggering lysis by natural killer cells [55]	6.92	0.0425
Fcgr4	Promotes macrophage-mediated phagocytosis and antigen presentation to T cells [56]	10.03	0.0307
Ifi204	Acts as a nuclear innate DNA sensor resulting in inflammasome activation [57]	15.33	0.0202
Il1rn	Binds non-productively to the interleukin-1 receptor preventing IL-1 from sending a signal [58]	48.31	0.0017
Irgm1	Involved in the polarization of M1 [inflammatory driven] macrophages [45]	8.36	0.0303
Lilrb3	Functions as an inhibitory receptor to help balance the function of innate immune cells [47]	10.74	0.0062
S100a9	Controls macrophage accumulation and cytokine production [59]	17.85	0.0179
Slamf7	Induces B cell proliferation [50]	29.00	0.0425
Socs1	Involved in the negative feedback regulation of cytokine signaling [60]	6.98	0.0208
Tyrobp	Activates signal transduction and plays a role in inflammation [61]	9.42	0.0468

The fold changes in transcriptional activities within MCMV-infected eyes of groups of healthy mice (n = 3), MAIDS-4 mice (n = 3), and MAIDS-10 mice (n = 3) were determined for all 561 differentially expressed immune response genes. The 15 genes for the MCMV-infected eyes showing the greatest fold change increase when compared with mock-infected eyes are summarized, with known function(s), peak expression, and *p* values.

**Fig 4:**
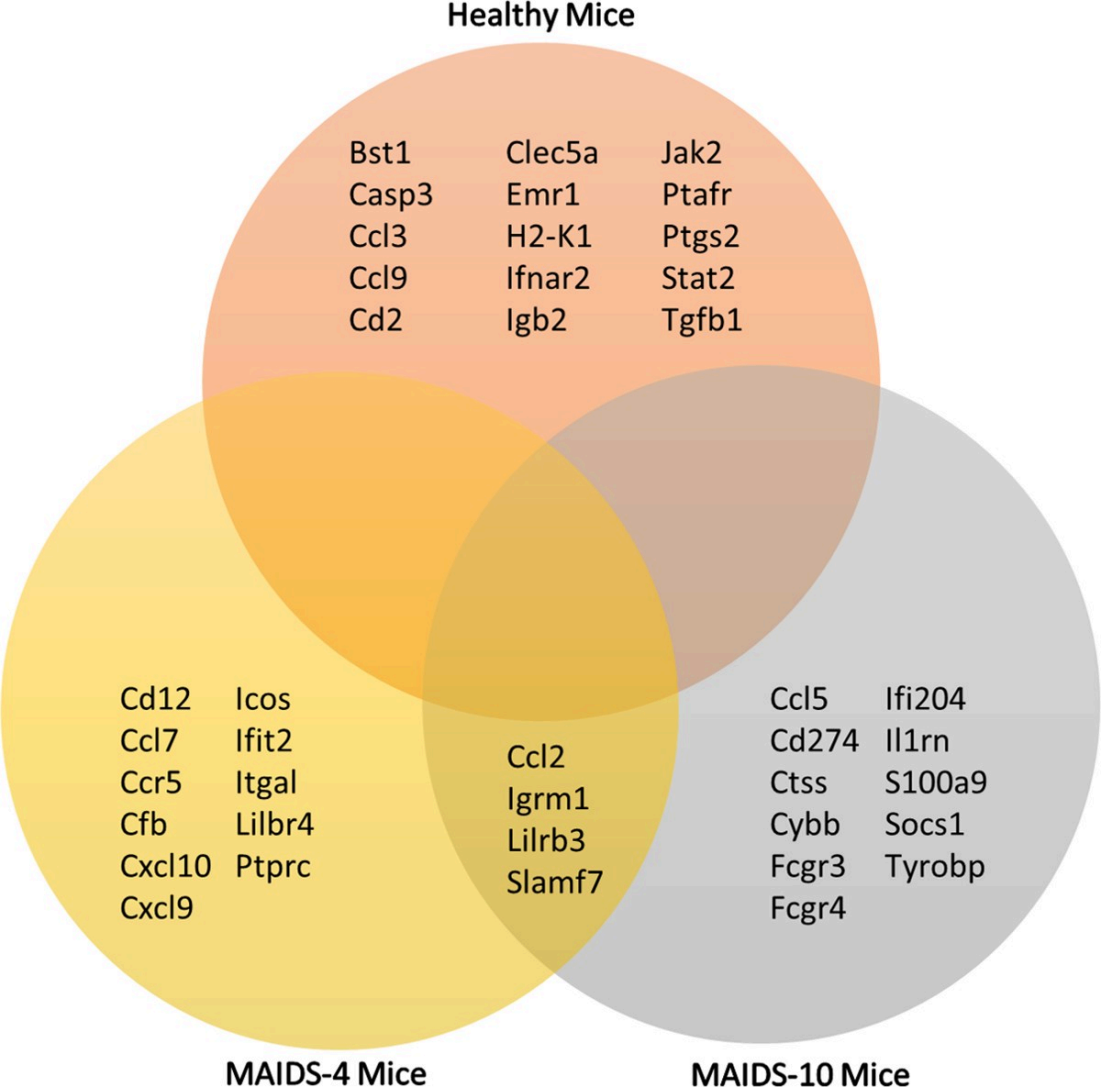
Venn diagram comparing the expression of 15 differentially expressed immune response genes showing the greatest upregulation of activity within MCMV-infected eyes of groups of healthy mice, MAIDS-4 mice, and MAIDS-10. Following analysis of the differentially expressed transcriptional activity for 561 immune response genes within MCMV-infected eyes of groups of healthy mice (n = 3), MAIDS-4 mice (n = 3), and MAIDS-10 mice (n = 3), those 15 genes showing the greatest upregulation of activity at all times examined postinfection (see Table 1) were compared for possible, shared activities among the three animal groups. While none of the genes presented shared activities among all three groups, of the 15 most active genes, 4 genes were found to be commonly expressed within the MCMV-infected eyes of MAIDS-4 mice and MAIDS-10 mice.

A detailed comparison of the top 15 immune response genes activated within MCMV-infected eyes of healthy mice, MAIDS-4 mice, and MAIDS-10 mice also revealed that some upregulated gene activities were exclusive to each animal group whereas other upregulated gene activities were shared between and among groups. This is depicted in the Venn diagram shown in Fig 4. Whereas the top 15 upregulated genes of the MCMV-infected eyes of healthy mice that are absolutely resistant to MCMV retinitis development were found to be exclusive to this animal group, while 4 of the top 15 upregulated genes of the eyes of MCMV-infected mice of MAIDS-4 and MAIDS-10 groups were shared between and among the animal groups. This observation, however, should not diminish the observation that 11 of the top 15 upregulated genes of the MCMV-infected eyes of these animal groups were exclusive to MAIDS-4 mice and MAIDS-10 mice, animals that exhibited remarkably distinct patterns of MCMV-induced retinal disease [17].

### Analysis of 14 additional immune response gene transcripts of MCMV-infected eyes of healthy mice, MAIDS-4 mice, and MAIDS-10 mice associated with programmed cell death pathways

Use of the commercially available NanoString nCounter Murine Immunology Panel provided a wealth of new and, at times, unexpected changes in the expression of 561 immune response genes during the onset and development of MAIDS-related MCMV retinitis. Given our present interest in programmed cell death pathways and their relative roles in the pathogenesis of MCMV-induced full-thickness retinal necrosis in MAIDS-10 mice [62,63], we created a custom panel consisting of 14 genes. This panel included 3 genes associated with autophagy, 3 genes associated with necroptosis, 2 genes associated with parthanatos, and 6 genes associated with pyroptosis and inflammasomes. Importantly, gene transcription analysis using this custom gene panel for cell death pathways was performed using the same samples collected from MCMV-infected eyes of healthy mice, MAIDS-4 mice, and MAIDS-10 mice that were earlier used to generate data using the commercially available murine immunology gene panel. Genes associated with apoptosis were excluded from this custom panel because we have already determined previously, using mice with MAIDS deficient in key apoptosis-associated genes, that this programmed cell death pathway contributes only minimally to the pathogenesis of MAIDS-related MCMV retinitis [62].

A summary of the upregulation or downregulation for each of the 14 immune response genes within MCMV-infected eyes for each animal group at 3, 6, and 10 days after intraocular MCMV inoculation is shown in Table 2. The overall positive (upregulated) or negative (downregulated) fold changes at peak expression including their *p* values of these response genes when compared with mock-infected eyes is also shown. Owing to the relatively small number of 14 genes being analyzed in this experiment, a two-fold change in gene activity was not used to exclude some genes for analysis as was done for the 561 immune response genes analyzed above. The MCMV-infected eyes of healthy mice which are absolutely resistant to the development of MCMV retinitis [17] exhibited a pattern of cell death pathway-associated gene activities that suggested significant quiescence of activity for each pathway at all days postinfection examined. Indeed, downregulation of gene activity was consistently observed for all 3 necroptosis genes and all 6 pyroptosis and associated inflammasome genes at 3, 6, and 10 days postinfection. Gene activities for autophagy and parthanatos were also found to be either downregulated or only minimally upregulated (< 2-fold increase) within MCMV-infected eyes of healthy mice at all days postinfection examined. As MCMV-infected eyes of animals at different stages of MAIDS development became more susceptible to the development of retinal disease at different stages of MAIDS development, however, genes associated with some, but not all, cell death pathways under investigation became increasingly active. This was apparent within the MCMV-infected eyes of MAIDS-4 mice and MAIDS-10 mice for necroptosis and pyroptosis and pyroptosis-associated inflammasomes but not for autophagy and parthanatos.

**Table 2:** Summary of differentially expressed immune response genes associated with programmed cell death pathways within MCMV-infected eyes of groups of healthy mice, MAIDS-4 mice, and MAIDS-10 mice.

Fold Change of MCMV-infected Eyes of Healthy Mice							
Cell Death Pathway	Genes	Day 3		Day 6		Day 10	
		Δ	p value	Δ	p value	Δ	p value
Autophagy	Atg12	1.07	0.3855	1.24	0.0792	1.14	0.1344
Autophagy	Atg5	-1.05	0.0155	-1.13	0.0269	-1.02	0.0047
Autophagy	Becn1	0.05	0.5346	-0.05	0.5090	1.07	0.3855
Necroptosis	Mlkl	-3.53	0.1688	-1.59	0.0012	-4.34	0.0500
Necroptosis	Ripk1	-1.46	0.0491	-1.31	0.0248	-1.63	0.0048
Necroptosis	Ripk3	-2.34	0.0983	-2.01	0.0632	-2.87	0.0016
Parthanatos	Parg	1.07	0.1772	1.24	0.2209	0.02	0.5158
Parthanatos	Parp1	0.07	0.5445	1.33	0.0385	1.11	0.1695
Inflammasome/Pyroptosis	Aim2	-2.27	0.0457	-1.45	0.0013	-2.07	0.0073
Inflammasome/Pyroptosis	Casp11	-6.83	0.2365	-2.86	0.0683	-7.90	0.1394
Inflammasome/Pyroptosis	Gsdmd	-2.64	0.0437	-1.95	0.0636	-2.91	0.0033
Inflammasome/Pyroptosis	Nlrc4	-3.78	0.1043	-1.81	0.1011	-4.14	0.0538
Inflammasome/Pyroptosis	Nlrp1b	-0.01	0.5154	-0.07	0.5284	-1.24	0.0510
Inflammasome/Pyroptosis	Nlrp3	-2.42	0.0483	-0.01	0.5328	-2.25	0.0837
Fold Change of MCMV-infected Eyes of MAIDS-4 Mice							
Cell Death Pathway	Genes	Day 3		Day 6		Day 10	
		Δ	p value	Δ	p value	Δ	p value
Autophagy	Atg12	0.05	0.5378	-1.13	0.0314	-0.44	0.5109
Autophagy	Atg5	1.52	0.2518	1.19	0.0172	0.10	0.6342
Autophagy	Becn1	1.25	0.3402	1.19	0.0855	0.05	0.5810
Necroptosis	Mlkl	4.73	0.1322	6.69	0.0625	6.01	0.2067
Necroptosis	Ripk1	1.98	0.1282	2.25	0.1306	2.34	0.0547
Necroptosis	Ripk3	3.18	0.2738	4.49	0.2026	5.29	0.0467
Parthanatos	Parg	-0.03	0.5322	-0.13	0.5056	-1.28	0.0612
Parthanatos	Parp1	1.06	0.5000	1.21	0.1669	-1.49	0.1188
Inflammasome/Pyroptosis	Aim2	2.56	0.0470	2.72	0.1099	3.03	0.0995
Inflammasome/Pyroptosis	Casp11	8.74	0.1416	11.25	0.0666	8.81	0.0533
Inflammasome/Pyroptosis	Gsdmd	3.59	0.0783	5.05	0.1068	3.59	0.1095
Inflammasome/Pyroptosis	Nlrc4	7.67	0.1999	7.40	0.0953	4.61	0.0291
Inflammasome/Pyroptosis	Nlrp1b	1.58	0.2863	1.74	0.1732	4.54	0.2836
Inflammasome/Pyroptosis	Nlrp3	4.23	0.1047	3.41	0.0672	3.64	0.1983
Fold Change of MCMV-infected Eyes of MAIDS-10 Mice							
Cell Death Pathway	Genes	Day 3		Day 6		Day 10	
		Δ	p value	Δ	p value	Δ	p value
Autophagy	Atg12	-0.07	0.5202	1.19	0.3286	1.08	0.0424
Autophagy	Atg5	1.32	0.0101	1.36	0.1665	1.69	0.0092
Autophagy	Becn1	1.41	0.0465	1.28	0.2578	1.59	0.1850
Necroptosis	Mlkl	5.75	0.1482	4.16	0.1853	3.43	0.1166
Necroptosis	Ripk1	2.20	0.0978	1.96	0.0565	2.17	0.0272
Necroptosis	Ripk3	3.80	0.0057	3.93	0.0228	4.14	0.0091
Parthanatos	Parg	-1.14	0.0119	1.21	0.2048	-1.26	0.0085
Parthanatos	Parp1	-0.13	0.5111	1.19	0.1190	-1.63	0.0736
Inflammasome/Pyroptosis	Aim2	2.08	0.1594	2.44	0.0244	2.04	0.1032
Inflammasome/Pyroptosis	Casp11	12.65	0.0358	5.07	0.1811	5.83	0.0631
Inflammasome/Pyroptosis	Gsdmd	4.41	0.0707	2.68	0.1036	3.63	0.0073
Inflammasome/Pyroptosis	Nlrc4	7.20	0.2324	6.05	0.3290	4.23	0.0659
Inflammasome/Pyroptosis	Nlrp1b	-0.10	0.5529	1.80	0.3369	1.12	0.3362
Inflammasome/Pyroptosis	Nlrp3	3.08	0.0882	3.36	0.1534	1.40	0.2788

The fold changes in transcriptional activities within MCMV-infected eyes when compared with mock-infected eyes of groups of healthy mice (n = 3), MAIDS-4 mice (n = 3), and MAIDS-10 mice (n = 3) were determined for genes associated with autophagy, necroptosis, parthanatos, and pyroptosis including inflammasomes at 3, 6, and 10 days postinfection.

## Discussion

Herein we have confirmed and extended our understanding of some of the immunologic events that take place during the onset and development of cytomegalovirus retinitis in the unique setting of retrovirus-induced immunosuppression. This was accomplished by performing a comprehensive transcriptional analysis of immune response genes during the pathogenesis of MAIDS-related MCMV retinitis, a well-characterized, reproducible, and clinically relevant mouse model of AIDS-related HCMV retinitis [13]. Our findings show that (i) the pathogenesis of retinal disease in MCMV-infected eyes of MAIDS-10 mice susceptible to full-thickness retinal necrosis development is associated with the robust, differential expression of extensive number of immune response genes that operate in several distinct immune response pathways; (ii) the temporal development of MCMV retinitis within the eyes of MAIDS-10 mice is a dynamic process that involves both the upregulation and downregulation of several immune response genes at different times after intraocular MCMV infection; and (iii) the pattern of immune response gene activation differs remarkably within MCMV-infected eyes of healthy mice resistant to retinitis development when compared with MCMV-infected eyes of mice at different stages of MAIDS that exhibit a profound difference in their susceptibility to full-thickness retinitis development. A more focused companion investigation also provided compelling evidence for the stimulation of the transcription of multiple genes associated with the necroptosis and pyroptosis programmed cell death pathways during the development MAIDS-related MCMV retinitis.

For our investigation of immune response gene expression during the pathogenesis of MAIDS-related MCMV retinitis, we used the NanoString nCounter assay. This recently developed technology platform is capable of making without amplification a direct multiplexed measurement of gene expression within the ocular compartment following intraocular MCMV inoculation of mice for comparisons during immunocompetence and MAIDS without recourse to any need for sequence amplication. This amplification-free microarray technology is a powerful tool that allows for the simultaneous quantification of the expression of hundreds of genes at several distinct time points during disease pathogenesis. Even so, we initiated this investigation mindful of several limitations that this experimental approach enjoys. Firstly, the amount of data obtained after the performance of a single experiment is overwhelming and requires thoughtful use of statistical analysis to help provide meaningful conclusions [64]. Secondly, subsequent performance of quantitative RT-PCR and/or western blot assays is needed to confirm the upregulation or downregulation of an individual gene under investigation. Thirdly, the data generated are descriptive and often do not provide mechanistic insights into the function of an individual gene or its functional gene product during a pathogenic event. Finally, it has not escaped our attention that the NanoString nCounter assay provides only fold-change differences in transcription activity that may or may not be biologically significant to disease pathogenesis in that the quantitative fold-change in activity of an individual gene may not be a true reflection of the unique kinetics of that particular gene’s mRNA or its gene product and consequently pertinent changes in its functional ability. For example, a two-fold increase in transcriptional activity may be biologically significant for one gene, but another gene may require a far greater transcriptional increase for a greater duration and at a particular time to be biologically significant.

Despite these perceived limitations, the NanoString nCounter assay has proven to be a useful and powerful tool to point us quickly in new directions of investigation and thereby expand our knowledge base of a series of immune functions that contribute (or do not contribute) to the onset of MCMV-induced retinal disease and progression to full-thickness necrosis during MAIDS. Use of the NanoString nCounter assay has also provided the opportunity to confirm previous findings by us and others on the role of various immune responses toward the pathogenesis of experimental MCMV retinitis during immunosuppression. Most of the findings presented herein are in agreement with findings documented by us in past publications that have focused on various immune-mediated pathways and/or individual molecules associated with innate or adaptive immunity vis-a-vis the pathogenesis of MAIDS-related MCMV retinitis. These include roles for humoral immunity [65], cellular immunity [66,67], suppressor of cytokine signaling (SOCS) pathways [60,68], TNF-α [17], interferon-γ [17], and a number of proinflammatory cytokines associated with innate immunity [62,69] as summarized by us in a recent review [16]. The use of a custom panel consisting of a smaller subset of genes associated with necroptosis and pyroptosis as well as pyroptosis-related inflammasomes in this report also served to confirm our previous determination that these programmed cell death pathways appear to be involved in the progression of MCMV retinitis during MAIDS [62] as evidenced by this earlier report of significant intraocular stimulation of necroptosis-associated receptor interacting protein kinase 3 (RIPK3), pyroptosis-asssociated caspase-1, interleukin-1β, interleukin-18, and the AIM2 inflammasome.

Careful inspection of the data obtained in the present investigation also exposed an unexpected difference in findings reported herein using the NanoString technology and a publication by us for another programmed cell death pathway, parthanatos, and MAIDS-related MCMV retinitis [63]. Whereas the transcription levels of *parg* and *parp1*, two genes associated with the parthanatos pathway [70–75], show negligible activity within MCMV-infected eyes of MAIDS-10 at either 3, 6, or 10 days after intraocular MCMV inoculation in the present investigation, we have documented previously a significant increase in *parp1* mRNA and protein production as well as *parg* mRNA and protein synthesis by quantitative RT-PCR and western blot assays, respectively, at the same times examined after intraocular MCMV inoculation of MAIDS-10 mice [63]. The reason for this major discrepancy in findings remains unclear but may be due to differences in the regional specificity of probes used by the NanoString nCounter assays [18]. That these parthanatos-associated genes are indeed upregulated is undeniable given the marked intraocular stimulation of protein production previously shown by us for their respective gene products [63]. These apparently conflicting observations, however, only serve to underscore the need to confirm all NanoString nCounter data using further quantitative RT-PCR and/or western blot analysis for each gene of interest.

In summary, our experience using the NanoString nCounter assay to provide a novel transcriptional analysis of 575 immune response genes within MCMV-infected eyes of mice at different stages of MAIDS development compared with MCMV-infected eyes of immunologically normal mice has provided new, and at times, unexpected information on the pathogenesis of MAIDS-related MCMV retinitis. By extension, our findings may also improve our understanding of the pathogenesis AIDS-related HCMV retinitis as well as other AIDS-related opportunistic virus infections. While helpful in many ways, we have also identified areas of caution when using this powerful research tool. Future use of this technology by us will be directed toward the identification of host genes expressed by different cell populations of retinal tissues during the onset and progression of MAIDS-related MCMV retinitis. Moreover, because host RNA constitutes an overwhelming portion of the total RNA recovered from infected tissue samples when compared with pathogen RNA which usually comprises a vanishingly small portion of total RNA [76], use of this technology will also allow us to investigate with greater precision the pattern of mRNA synthesis for individual MCMV genes that are expressed within retinal tissues and retina-related cell populations during the course of disease development following intraocular MCMV infection of retrovirus-immunosuppressed mice.

## Materials and methods

### Viruses

Stocks of MCMV (Smith) were propagated through salivary glands of groups of BALB/c mice (Harlan Laboratories, USA) as described previously [13]. Briefly, fourteen days following intraperitoneal injection of 10^2^ to 10^3^ plaque forming units (PFU) of MCMV contained within 0.2 ml volume, salivary glands were harvested aseptically, pooled, and homogenized (10% [wt/mol]) in Dulbecco’s modified eagle media (DMEM, Corning Life Sciences, Manassas, VA, #10–013). Virus stocks were clarified by centrifugation, aliquoted, and stored in liquid nitrogen prior to quantification by standard plaque assay on monolayers of mouse embryonic fibroblasts as described previously [13]. A fresh aloquot of MCMV stock was thawed and used for each experiment.

Stocks of murine retrovirus (LP-BM5 murine leukemia virus [MuLV]) were prepared in monolayers of SC-1 fibroblasts (ATCC #CRL-1404) and SC-1/MuLV LP-BM5 cells [77] kindly provided by the AIDS Research and Reference Reagent Program, Division of AIDS, NIAID, NIH (Germantown, MD). Six days following seeding at a 1:1 ratio, the cells were scraped into SC-1 media and stored at -80°C. Prior to use, LP-BM5 stocks were thawed and clarified by centrifugation to remove cellular debris.

### Animals

Adult female wildtype BALB/c mice used for the preparation of MCMV stocks were purchased from Harlan Laboratories (Indianapolis, IN, USA). Adult female wildtype C57BL/6 mice used for all MAIDS studies were purchased from Jackson Laboratory (Bar Harbor, ME, USA). All mice were maintained on alternative 12-hr light/dark cycles and allowed unrestricted access to food and water. All procedures were conducted with strict compliance to National Institutes of Health and the Association for Research in Vision and Ophthalmology (ARVO) statement for Use of Animals in Ophthalmic and Vision Research guidelines and in accordance with Georgia State University Institutional Animal Care and Use Committee (IACUC) approved protocols.

### Induction of MAIDS

MAIDS was induced by injecting 1.0 ml of inoculum containing approximately 5 × 10^3^ to 5 × 10^4^ infectious LP-BM5 murine leukemia retrovirus into the peritoneum of C57BL/6 mice. Mice with MAIDS of 4 weeks’ duration (MAIDS-14 mice) and 10 weeks’ duration (MAIDS-10 mice) were used throughout the investigation and compared with age-matched healthy C57BL/6 mice.

### Experimental mouse model of MCMV retinitis

Details for the MAIDS model of MCMV retinitis used throughout the investigation have been described by us previously [13,16,62]. Briefly, the left eyes of groups of healthy mice, MAIDS-4 mice, and MAIDS-10 mice were subjected to intraocular (subretinal) inoculation with approximately 10^4^ PFU of MCMV contained within a 2-ul volume of DMEM. The right contralateral eyes of all mice were inoculated intraocularly with DMEM and served as controls.

### NanoString nCounter assay

Whole MCMV-infected and control eyes were collected from all animal groups at 3, 6, and/or 10 days after intraocular inoculation and stored at 4C in RNAlater solution (Ambion, Austin, TX) prior to NanoString nCounter analysis. At time of analysis, eyes were individually homogenized in 1.0 ml of TRIzol reagent (Invitrogen Life Technologies, Carlsbad, CA) using a 2-ml Ten Broeck tissue grinder (Wheaton, Millville, NJ). Total RNA was extracted in chloroform and purified using the PureLink RNA Mini Kit according to the manufacturer’s instructions (Ambion/ThermoFisher, Grand Island, NY). The extracted RNA from each group and their respective time points were pooled and the RNA concentrations were determined using a Nanodrop 2000 spectrophotometer (Thermo Scientific, Pittsburgh, PA). Approximately 100 ng of the purified total RNA from each time point of each animal group were analyzed using the nCounter Analysis System (NanoString Technologies, Seattle, WA) according to the manufacturer’s instructions in combination with the Murine Immunology Panel which contained 561 unique RNA barcodes. Probes for 15 internal and housekeeping genes such as ribosomal protein L10, beta-actin, beta-2-microglobuin, glyceraldehyde 3-phosphate dehydrogenase, and ribosomal protein L19 were incorporated into the NanoString codesets of this panel. A second custom-made panel that included 14 unique genes not included in the Murine Immunology Panel but associated with programmed cell death pathways as well as three housekeeping genes was used in another set of studies. Analysis of raw mRNA data was performed using the NanoString nSolver™ analysis software version 4.0.

### Statistical analysis

Two independent experiments were performed for each study and were run independently through the NanoString nSolver™ analysis software. All *p* values were determined on fold changes of raw mRNA counts as determined by the NanoString nSolver™ software and performed with a significance level (α) set to 0.05; *p* values of < 0.05 were considered statistically significant. Statistical analysis were performed by comparing MCMV-infected eyes with mock-infected eyes (controls) by unpaired, two-sided Student’s *t*-test.

## Supporting information

S1 Fig Number of upregulated immune response genes within MCMV-infected eyes of groups of healthy mice, MAIDS-4 mice, and MAIDS-10 mice when categorized according to more focused immunologic pathways. Transcriptional activity of immune response genes of MCMV-infected eyes of groups of healthy mice (n = 3), MAIDS-4 mice (n = 3), and MAIDS-10 mice (n = 3) with a fold change of greater than two when compared with mock-infected eyes at 3, 6, or 10 days postinfection were categorized into more focused NanoString-defined immunologic pathways that exhibited the robust upregulation. Immunologic pathways included those associated with (A) TLR Signaling (B)Phagocytosis and Degradation, (C) NOD-like Receptor (NLR) Signaling, (D) Type I Interferon (IFN) Signaling, and (E) Type II Interferon (IFN) Signaling, (F) Tumor Necrosis Factor (TNF) Signaling, (G) Chemokine Signaling, (H) NF-kB Signaling, and (I) MHC Class I Antigen Presentation.
(TIF)

S1 Table Summary of all 561 immune defense genes analyzed for MCMV-infected eyes of groups of healthy mice (n = 3), MAIDS-4 mice (n = 3), and MAIDS-10 mice (n = 3) at 3, 6, and 10 days postinfection showing fold-change expression together with *p* values when compared with mock-infected eyes. Variability between individual eyes per group is not shown due to pooling of individual eyes.
(PDF)
