# Supplementary material for: Transcriptional analysis of immune response genes during pathogenesis of cytomegalovirus retinitis in mice with murine acquired immunodeficiency syndrome
Source: PLoS Pathog. 2020 Nov 6;16(11):e1009032. doi: 10.1371/journal.ppat.1009032 (PMC7647057; doi:10.1371/journal.ppat.1009032)
Supplement: S1 Table — Variability between individual eyes per group is not shown due to pooling of individual eyes. (PDF) [file ppat.1009032.s002.pdf]

Gene	Healthy						MAIDS-4						MAIDS-10					
	Day 3		Day 6		Day 10		Day 3		Day 6		Day 10		Day 3		Day 6		Day 10	
	Fold Change	p value	Fold Change	p value	Fold Change	p value	Fold Change	p value	Fold Change	p value	Fold Change	p value	Fold Change	p value	Fold Change	p value	Fold Change	p value
Abcb10	-0.055	0.5176	-1.325	0.0641	-0.235	0.5000	1.71	0.4816	1.05	0.9882	-1.17	0.0088	-1.285	0.0680	1.015	0.2048	-1.315	0.0151
Abcb1a	-1.025	0.0016	0.195	0.6323	-0.105	0.5085	-1.22	0.0201	0.245	0.6640	0.12	0.5972	0.08	0.5731	1.04	0.2952	-1.320	0.0329
Abcf1	0.115	0.5757	0.045	0.5810	-1.41	0.0658	1.12	0.5000	1.465	0.4860	1.34	0.4386	1.16	0.2952	1.12	0.4097	-1.150	0.0030
Abl1	-1.05	0.0124	-1.23	0.0029	0.04	0.5345	-0.015	0.5646	1.63	0.4845	1.08	0.1560	-1.44	0.0078	-0.02	0.5297	-0.255	0.5197
Adal	-1.38	0.0214	-1.675	0.0202	-1.57	0.0544	-1.625	0.0496	-0.26	0.5050	-0.44	0.5489	-1.845	0.0413	-2.11	0.0164	-3.130	0.0462
Ahr	1.675	0.1963	1.045	0.3228	1.245	0.0647	1.54	0.1725	-1.19	0.0377	1.17	0.4603	1.35	0.0363	0.025	0.5251	-0.020	0.5062
Aicda	-1.76	0.0644	-1.27	0.0392	-1.345	0.0095	0.17	0.6383	4.995	0.3565	-0.205	0.5278	-1.65	0.0503	1.25	0.2639	-2.710	0.1724
Aire	-0.82	0.6123	3.6	0.3106	-1.94	0.1198	-0.18	0.6506	31.84	0.4711	1.08	0.9789	2.17	0.3352	1.515	0.4165	1.175	0.4405
Alas1	1.075	0.4546	-0.025	0.5513	-1.16	0.0059	0.01	0.5187	1.72	0.0529	-0.185	0.5795	1.315	0.0504	1.36	0.1560	-1.090	0.0244
App	-0.025	0.5268	-1.14	0.0030	-0.01	0.5062	1.08	0.3556	0.055	0.5552	1.09	0.1392	1.155	0.2869	1.1	0.2422	1.100	0.3440
Arhgdib	2.81	0.2994	3.07	0.3931	3.365	0.1820	2.395	0.0478	3.145	0.3075	2.995	0.1336	3.035	0.1281	2.74	0.1264	6.140	0.1830
Atg16l1	1.05	0.0704	-1.04	0.0062	-1.205	0.0159	-0.035	0.5000	1.495	0.4801	-1.615	0.0377	-1.335	0.0150	-1.21	0.0288	-1.500	0.0407
Atm	1.32	0.0987	1.245	0.3949	-0.105	0.5222	1.18	0.3492	2.46	0.2557	-0.15	0.5082	0.185	0.6263	1.095	0.1638	0.020	0.5480
B2m	3.81	0.2683	2.57	0.4231	5.205	0.2820	4.545	0.2102	4.255	0.4225	2.895	0.3747	6.15	0.1504	2.815	0.1793	6.175	0.0619
Batf	2.155	0.2098	2.805	0.3314	1.985	0.0740	2.92	0.4054	4.1	0.1550	1.76	0.8550	2.25	0.1595	3.505	0.0317	3.335	0.0530
Batf3	-0.36	0.5395	0.075	0.6187	-1.58	0.0049	-1.495	0.0242	3.19	0.4310	2.12	0.2767	-1.44	0.0026	-1.235	0.0185	-2.925	0.1241
Bax	1.585	0.2708	1.715	0.2934	1.155	0.1799	1.865	0.0404	1.44	0.3066	1.78	0.1750	1.86	0.2892	1.525	0.2370	2.250	0.0810
Bcap31	0.01	0.5217	1.13	0.3145	0.06	0.5411	1.035	0.2578	-0.13	0.5191	1.065	0.0489	0.055	0.5496	0	0.5094	1.305	0.0313
Bcl2	-1.25	0.0339	-0.07	0.5202	-1.325	0.0068	0.535	0.8215	1.625	0.2972	-3.87	0.2904	-1.33	0.0246	-1.24	0.0510	-1.710	0.0235
Bcl3	2.695	0.3034	3.285	0.3935	1.915	0.3572	4.34	0.1168	5.385	0.1694	2.505	0.4246	3.87	0.2578	2.755	0.0163	2.085	0.0029
Bcl6	2.04	0.1032	1.505	0.2977	1.3	0.4781	4.92	0.1421	2.845	0.1277	-0.175	0.6342	2.49	0.1183	1.565	0.1493	1.585	0.2048
Bid	1.985	0.0355	1.77	0.2507	1.5	0.4056	2.19	0.0640	3.06	0.2030	0.385	0.7808	1.72	0.0879	2.06	0.1012	2.300	0.0294
Blnk	1.58	0.0329	1.235	0.0675	2.195	0.1082	1.775	0.0123	1.925	0.3876	1.84	0.0604	1.355	0.1496	1.65	0.4235	2.795	0.1547
Bst1	3.245	0.0298	2.38	0.2726	1.9	0.0564	4.52	0.0307	5.05	0.2147	3.01	0.2811	4.075	0.0403	3.67	0.2260	5.100	0.2555
Bst2	2.965	0.2222	3.385	0.3468	4.465	0.2852	5.04	0.0047	4.855	0.2637	2.555	0.2911	8.885	0.1463	3.35	0.2328	4.290	0.1186
Btk	1.86	0.3777	2.285	0.4195	1.275	0.2716	2.12	0.1980	3.535	0.1324	2.4	0.2730	1.555	0.0172	1.77	0.0903	1.850	0.0893
Btla	0.045	0.5346	1.7	0.4405	1.135	0.2048	-1.24	0.0142	4.39	0.3365	2.425	0.3067	-1.46	0.0207	1.265	0.2607	-1.270	0.0698
Btnl1	-1.215	0.0302	-1.25	0.0452	-1.85	0.0535	0.325	0.7521	1.98	0.2899	0.075	0.6143	-2.185	0.0010	-0.14	0.5083	-4.405	0.0088
Btnl2	-1.79	0.1217	2.25	0.3836	-2.065	0.1100	1.27	0.9687	27.52	0.4550	1.56	0.8782	1.44	0.4003	0.12	0.6022	-1.335	0.0612
C1qa	-0.085	0.5226	2.065	0.3596	3.995	0.1672	1.33	0.2554	3.545	0.2544	3.065	0.0630	1.88	0.0217	1.815	0.2483	4.295	0.1231
C1qb	1.27	0.3743	2.64	0.3625	5.345	0.2692	1.75	0.0592	2.875	0.0119	3.45	0.0052	1.58	0.0219	1.79	0.2024	5.620	0.0207
C1qbp	1.1	0.4296	-1.075	0.0230	-1.125	0.0255	0.01	0.5306	0.915	0.9737	1.02	0.9937	-1.065	0.0108	1.03	0.2048	-1.030	0.0063
C1ra	2.755	0.1478	1.76	0.4456	2.61	0.2904	3.705	0.0784	3.115	0.3182	0.435	0.8028	2.41	0.1421	2.23	0.1026	2.980	0.0989
C1s	1.945	0.1926	1.64	0.4222	2.77	0.1852	2.415	0.1649	3.45	0.0750	0.75	0.9149	1.815	0.0584	1.89	0.1270	3.320	0.0329
C2	1.82	0.0849	0.29	0.6878	1.655	0.1842	2.455	0.0676	2.91	0.0861	1.255	0.1589	1.835	0.0038	1.705	0.2970	2.010	0.2124
C3	4.54	0.2908	2.085	0.7853	3.995	0.2213	4.535	0.0921	10.205	0.1246	3.265	0.2468	4.035	0.0617	3.15	0.1027	4.860	0.0804
C4a	-0.055	0.5232	-0.075	0.5440	1.73	0.2557	1.135	0.4492	1.405	0.1761	1.395	0.2919	1.265	0.2607	-0.055	0.5000	0.140	0.6066
C4bp	1.345	0.5000	2.45	0.1508	-0.16	0.5527	6.15	0.6359	24.43	0.4372	3.275	0.4599	0.865	0.9644	0.005	0.5156	1.475	0.4792
C6	-1.325	0.0014	0.08	0.5650	-1.505	0.0089	0.47	0.8128	3.56	0.3466	0.235	0.6646	-1.68	0.0166	-1.165	0.0279	-2.440	0.0240
C7	-1.545	0.0784	0.175	0.6153	-2.455	0.2395	1.315	0.9286	6.345	0.4841	2.075	0.4817	-0.445	0.5065	-1.495	0.0319	-2.690	0.1781
C8a	2.015	0.2086	1.265	0.3369	-5.155	0.1566	5.005	0.7252	8.39	0.4646	-1.545	0.5320	1.485	0.8991	0.33	0.7228	1.930	0.4362
C8b	-1.425	0.0275	0.165	0.6239	-1.78	0.0999	-1.22	0.0115	2.23	0.2369	1.055	0.9834	-1.815	0.0011	-0.06	0.5830	-3.895	0.1038
C8g	-0.125	0.5084	-1.095	0.0106	-1.815	0.0102	0.17	0.6671	2.935	0.3955	1.93	0.4151	-1.305	0.0372	-0.115	0.5570	-2.080	0.0536
C9	-1.065	0.0015	1.24	0.3160	-1.97	0.0873	-0.225	0.5201	2.245	0.2276	2.725	0.4906	-2.055	0.0240	-0.295	0.5282	-3.635	0.0609
Camp	3.305	0.2687	1.91	0.3199	0.34	0.7630	2.025	0.3013	8.49	0.3432	3.835	0.4620	5.425	0.0495	4.19	0.2325	2.545	0.2268
Card9	3.745	0.4076	3.115	0.4356	1.88	0.2092	2.765	0.2724	7.045	0.2895	2.73	0.0331	3.555	0.0534	3.21	0.0944	3.600	0.0367
Casp1	4.37	0.2211	3.605	0.3603	2.86	0.0851	5.055	0.0243	7.45	0.0844	3.61	0.3153	4.655	0.1380	3.25	0.1284	5.985	0.1749
Casp2	0.01	0.5217	1.09	0.1392	-1.33	0.0137	-1.1	0.0242	1.365	0.0952	-2.325	0.2331	-1.415	0.0329	1.01	0.5000	-1.865	0.0189
Casp3	2.33	0.1183	2.155	0.2147	2.355	0.0117	3.925	0.1890	3.265	0.2249	-0.04	0.7508	3.375	0.0787	2.93	0.2186	3.705	0.1490
Casp8	2.455	0.1231	2.31	0.3962	1.805	0.0670	2.7	0.0486	2.615	0.3458	1.865	0.0769	2.98	0.0609	2.7	0.1257	2.580	0.0483
Ccbp2	-1.6	0.0635	-1.1	0.0030	-2.09	0.1359	-1.255	0.0633	3.445	0.4480	0.71	0.9051	-1.535	0.0163	1.07	0.3949	-3.255	0.1757
Ccl11	1.54	0.1166	0.205	0.6889	1.055	0.0577	1.44	0.1145	1.955	0.2440	-1.455	0.5533	1.79	0.0081	1.3	0.4028	1.500	0.2086
Ccl12	7.025	0.2562	5.135	0.3546	4.88	0.3736	10.88	0.0450	5.495	0.3612	2.92	0.2284	9.935	0.1493	4.425	0.2334	3.995	0.0835

Ccl19	1.73	0.0609	1.54	0.3904	1.2	0.1560	2.02	0.0436	2.92	0.3109	1.035	0.9891	1.94	0.2328	2.045	0.0638	1.785	0.0365
Ccl2	28.245	0.4291	8.145	0.4413	6.49	0.2956	51.23	0.0353	29.15	0.1584	6.55	0.1530	35.185	0.0263	6.93	0.3306	16.325	0.3516
Ccl20	-1.295	0.0319	-0.045	0.5318	-1.995	0.0117	-0.085	0.5836	1.72	0.3982	-1.65	0.0765	-1.705	0.0176	-0.11	0.5248	-4.955	0.0507
Ccl22	-1.205	0.0216	0.295	0.6867	-0.025	0.5181	1.88	0.3907	2.035	0.1711	0.795	0.9548	-0.32	0.5095	0.075	0.5535	1.055	0.2716
Ccl24	-2.16	0.0201	-0.275	0.5170	-2.285	0.0647	-0.635	0.5152	1.135	0.9679	0.155	0.6005	-1.925	0.0076	-1.385	0.0307	-2.900	0.0505
Ccl25	-1.235	0.0526	-0.04	0.5000	-1.41	0.0132	-1.515	0.0442	1.43	0.3241	-1.74	0.1636	-1.62	0.0146	-1.305	0.0262	-2.670	0.0398
Ccl26	-0.305	0.6356	1.725	0.1835	-1.595	0.0769	0.8	0.9443	19.8	0.4702	1.675	0.4704	1.555	0.3623	0.285	0.7225	-0.080	0.5611
Ccl3	6.945	0.2299	4.155	0.2323	2.275	0.0324	5.325	0.0653	4.665	0.3280	3.385	0.7385	5.685	0.1410	5.675	0.2393	3.020	0.1305
Ccl4	4.29	0.2328	3.3	0.3018	1.315	0.1678	4.085	0.1949	4.425	0.2414	3.045	0.4461	4.6	0.2837	4.03	0.1405	1.880	0.1762
Ccl5	70.11	0.4398	5.805	0.4288	15.865	0.4115	117.93	0.1218	16.87	0.4595	1.915	0.8159	83.315	0.0323	9.18	0.3332	25.545	0.3682
Ccl6	0.41	0.7873	2.395	0.3015	1.88	0.2156	1.29	0.1714	3.355	0.0713	3.105	0.1384	1.81	0.1906	2.545	0.0021	2.830	0.0139
Ccl7	19.975	0.4080	9.295	0.4406	13.495	0.3376	34.39	0.0350	21	0.4141	4.575	0.4512	36.635	0.2126	7.555	0.2973	23.485	0.1997
Ccl8	2.45	0.4269	3.18	0.4050	9.13	0.3116	2.435	0.0421	3.435	0.0896	5.98	0.3645	1.935	0.0510	1.48	0.2513	5.790	0.4035
Ccl9	2.74	0.3096	3.91	0.3980	3.085	0.0107	3.48	0.1118	3.705	0.1061	3.75	0.0531	3.665	0.0512	3.345	0.1759	5.275	0.1271
Ccr10	-0.27	0.5000	-1.14	0.0030	-1.81	0.1055	-0.05	0.5060	3.455	0.3864	1.875	0.4574	-1.63	0.0363	-1.18	0.0437	-3.215	0.0174
Ccr2	7.325	0.4174	3.6	0.4597	2.745	0.1860	7.075	0.1186	7.505	0.0367	3.02	0.4772	6.245	0.0575	2.995	0.0683	5.070	0.1266
Ccr3	-1.135	0.0134	-0.015	0.5062	-1.51	0.0101	1.125	0.0760	1.79	0.2097	-1.395	0.0833	-2.32	0.0383	-1.235	0.0071	-2.975	0.0726
Ccr4	0.01	0.5277	1.33	0.4248	-1.745	0.0290	1.25	0.9437	4.355	0.3959	1.335	0.0285	-1.51	0.0405	0.02	0.5534	-2.080	0.0165
Ccr5	5.89	0.3050	4.36	0.4434	3.325	0.0884	6.425	0.1223	11.095	0.0123	4.325	0.2986	6.565	0.0531	3.98	0.1670	5.935	0.1229
Ccr6	-1.46	0.0645	1.225	0.1526	-1.655	0.0491	0.26	0.7345	4.43	0.4648	1.055	0.0577	-1.91	0.0827	-1.145	0.0282	-3.570	0.2193
Ccr7	1.615	0.3175	2.45	0.3039	1.945	0.1360	2.16	0.3416	6.485	0.1299	3.21	0.4174	1.845	0.0861	2.875	0.0525	0.475	0.7863
Ccr8	-7.22	0.3828	-8.215	0.4023	-12.585	0.4289	14.65	0.6601	57.445	0.4989	-0.115	0.8703	-4.065	0.0570	-41.03	0.2146	-61.945	0.0044
Ccr9	-1.16	0.0088	-1.045	0.0109	-1.45	0.0441	0.355	0.7260	4.245	0.4197	-0.175	0.5841	-2.335	0.0048	-0.05	0.5675	-2.185	0.1330
Ccrl1	-0.03	0.5536	-1.17	0.0293	-1.38	0.0374	0.05	0.6204	1.815	0.4214	0.025	0.5892	-1.935	0.0894	-0.18	0.5000	-3.245	0.1091
Ccrl2	6.505	0.2405	3.175	0.3108	1.855	0.0557	6.765	0.1449	7.475	0.0143	5.755	0.2298	9.01	0.1475	4.625	0.2550	2.810	0.1518
Cd109	-1.84	0.0179	-1.505	0.1119	-1.215	0.0158	-1.81	0.0091	-0.13	0.5786	-0.25	0.5149	-1.87	0.0509	-1.735	0.0105	-1.795	0.1050
Cd14	3.615	0.0763	2.725	0.2310	1.63	0.2578	3.11	0.1510	2.865	0.2449	6.085	0.3968	4.85	0.0626	3.91	0.2564	2.465	0.2995
Cd160	0.055	0.6010	-0.035	0.5150	-2.47	0.0128	1.39	0.9116	2.64	0.2025	-0.965	0.5158	-1.24	0.0341	0.24	0.6611	-2.485	0.0646
Cd163	-1.66	0.0120	-1.565	0.0335	-1.72	0.0397	-0.275	0.5309	0.53	0.8253	0.01	0.5635	-1.5	0.0204	-1.745	0.0313	-3.330	0.1487
Cd164	1.305	0.0727	1.04	0.1560	-1.02	0.0016	1.23	0.2611	-1.125	0.0045	-1.13	0.0030	1.225	0.1257	0.045	0.5434	1.130	0.2338
Cd19	-1.38	0.0321	-1.09	0.0244	-1.275	0.0182	-1.21	0.0403	2.67	0.3176	-0.195	0.6108	-1.56	0.0472	1.18	0.0704	-1.855	0.0545
Cd1d1	-0.03	0.5180	0.295	0.7162	1.07	0.3949	-1.395	0.0728	1.83	0.2605	1.875	0.0832	-1.16	0.0471	1.295	0.2366	1.155	0.2869
Cd2	1.54	0.0936	3.53	0.4585	2.74	0.0365	1.975	0.2886	11.535	0.0992	3.33	0.4715	2.14	0.1586	3	0.1103	4.460	0.1129
Cd209g	-1.59	0.0442	-1.54	0.0150	-2.155	0.0252	-1.475	0.1008	3.09	0.4796	1.88	0.4535	-1.72	0.0234	-1.795	0.0216	-2.775	0.0547
Cd22	-1.265	0.0126	1.055	0.2716	-0.015	0.5000	-0.025	0.6666	2.44	0.4475	-0.085	0.6570	-1.57	0.0050	-0.07	0.5468	-1.615	0.1073
Cd226	0.495	0.8466	2.7	0.4727	3.165	0.4389	0.615	0.8586	9.88	0.1037	4.69	0.3598	3.09	0.0908	1.74	0.3602	3.620	0.1931
Cd244	2.125	0.3314	2.325	0.4927	1.05	0.4296	2.3	0.1304	9.14	0.2579	5.005	0.2980	1.735	0.2578	2.17	0.2194	1.480	0.1434
Cd247	-1.215	0.0158	2	0.3624	-1.065	0.0170	1.475	0.3999	3.01	0.2048	2.725	0.1186	1.3	0.2422	1.87	0.2863	1.445	0.0783
Cd24a	-0.12	0.5028	-1.315	0.0590	-0.08	0.5464	-1.845	0.0257	-6.955	0.3108	-3.035	0.2625	-1.37	0.0749	-1.885	0.0671	-1.300	0.0607
Cd27	1.28	0.2578	3.165	0.4389	1.55	0.1368	1.82	0.3486	9.865	0.1015	3.395	0.0989	1.655	0.1294	2.63	0.3212	1.955	0.2324
Cd274	4.61	0.2744	3.275	0.4040	2.87	0.0846	6.695	0.1058	14.29	0.0885	3.905	0.2615	7.355	0.0145	3.535	0.1980	4.035	0.2036
Cd28	-0.085	0.5000	0.135	0.6158	-1.56	0.0472	0.075	0.6230	2.645	0.2610	1.125	0.3053	-1.76	0.0369	0.015	0.6424	-3.170	0.1162
Cd34	-0.06	0.5059	-0.11	0.5085	0.005	0.5275	-1.355	0.0041	0.245	0.6640	-0.305	0.5048	-1.195	0.0304	-1.19	0.0464	1.115	0.0277
Cd36	-2.08	0.0269	-1.82	0.0316	-0.07	0.5229	-2.48	0.0602	-2.06	0.1273	2.29	0.2311	-1.285	0.0153	-1.44	0.0495	1.365	0.4912
Cd3d	1.41	0.2369	3.99	0.4357	4.115	0.2473	2.285	0.4874	6.11	0.4087	1.71	0.8456	2.535	0.0228	3.74	0.2083	6.605	0.0986
Cd3e	-1.24	0.0369	2.37	0.4632	1.76	0.2023	1.13	0.0972	4.89	0.1432	2.75	0.2639	1.34	0.3913	2.545	0.2827	1.940	0.2028
Cd3eap	-1.29	0.0128	-1.045	0.0140	-1.65	0.0456	0.9	0.9738	1.915	0.3471	-3.095	0.2924	-1.175	0.0161	0.07	0.5616	-2.220	0.0356
Cd4	-1.48	0.0690	0.125	0.5792	1.265	0.0599	-0.06	0.6123	4.365	0.3640	2.725	0.1042	-1.185	0.0277	1.265	0.4039	1.105	0.3072
Cd40	2.955	0.2487	2.575	0.3729	1.37	0.0515	3.86	0.0533	3.82	0.3836	0.36	0.8091	3.795	0.1465	3.145	0.2128	2.005	0.1098
Cd40lg	-1.11	0.0271	0.035	0.5430	-0.475	0.5043	-0.155	0.5893	3.75	0.3372	-0.81	0.5364	-1.475	0.0270	0.1	0.5718	-2.080	0.0883
Cd44	2.285	0.0910	1.695	0.3940	1.605	0.0368	2.49	0.0256	2.705	0.1485	2.11	0.2836	2.2	0.2330	2.02	0.0560	1.705	0.2750
Cd46	-0.12	0.7639	0.005	0.7575	-2.75	0.1109	-6.395	0.5395	19.38	0.4851	2.59	0.0200	-1.47	0.6300	2.065	0.4005	-0.105	0.5865
Cd48	3.15	0.2448	2.865	0.4067	3.68	0.0686	3.185	0.1774	3.265	0.4613	3.18	0.0871	2.935	0.1989	2.72	0.0591	5.060	0.0934
Cd5	-0.16	0.5238	2.185	0.4447	1.695	0.2158	3.18	0.4547	5.285	0.2943	0.88	0.9717	-1.245	0.0411	2.17	0.1850	1.860	0.2892
Cd53	2.34	0.1128	2.22	0.3509	-0.005	0.5244	1.57	0.3665	2.785	0.3100	2.47	0.0303	1.585	0.1750	2.085	0.0029	1.310	0.2869
Cd55	-1.37	0.0349	-0.215	0.5178	-1.455	0.0889	-0.16	0.5661	3.21	0.4912	1.51	0.2902	-1.045	0.0109	-1.33	0.0027	1.260	0.0731

Cd59b	-1.4	0.0292	-0.33	0.5186	-1.835	0.0415	-1.505	0.1020	3.11	0.4479	1.545	0.2392	-1.82	0.0563	-1.3	0.0798	-3.240	0.0614
Cd6	1.5	0.2086	3.095	0.4403	2.025	0.1727	0.515	0.8824	15.4	0.4228	2.765	0.0450	1.915	0.3471	2.865	0.2536	2.835	0.1870
Cd69	19.215	0.4483	4.775	0.4207	3.22	0.4320	31.24	0.3088	21.52	0.3006	4.49	0.1417	22.29	0.2890	3.805	0.1884	7.030	0.3090
Cd7	-0.04	0.5207	1.585	0.4471	-0.165	0.5054	-0.185	0.5517	3.71	0.2923	2.15	0.2081	-1.575	0.0111	1.475	0.4863	-1.635	0.1112
Cd74	1.99	0.0193	2.355	0.4212	5.93	0.3110	2.625	0.1029	4.59	0.1243	3.04	0.3408	3	0.0127	2.2	0.0371	5.465	0.0590
Cd79a	-1.39	0.0027	-1.2	0.0116	-1.62	0.1341	0.13	0.7063	3.36	0.3956	0.05	0.6772	-1.905	0.0839	1.08	0.5000	-2.510	0.0398
Cd79b	-0.17	0.5159	1.175	0.4813	-0.08	0.5387	1.385	0.4365	5.875	0.3994	3.525	0.3867	-1.675	0.0036	0.44	0.7683	0.070	0.5698
Cd80	2.03	0.1161	1.61	0.2386	1.315	0.3949	2.145	0.0527	2.61	0.1917	2.12	0.1980	1.59	0.2642	1.585	0.0054	1.385	0.1999
Cd81	-1.255	0.0099	-0.2	0.5026	1.09	0.2662	-1.13	0.0030	-1.49	0.0561	-1.12	0.0360	-1.19	0.0290	-1.41	0.0106	-0.170	0.5133
Cd82	-1.115	0.0105	-1.035	0.0109	0.015	0.5218	0.065	0.5501	-0.515	0.5042	0.005	0.5275	0.085	0.5735	1.025	0.1257	1.510	0.0125
Cd83	1.39	0.0489	1.495	0.1331	1.52	0.0610	1.68	0.2487	4.075	0.4225	0.07	0.6093	1.21	0.2829	1.11	0.0577	0.380	0.7534
Cd86	2.38	0.2533	1.95	0.4495	1.375	0.1738	3.465	0.1162	3.195	0.3479	2.545	0.0391	3.235	0.2547	2.41	0.0316	1.915	0.0521
Cd8a	-1.17	0.0235	1.945	0.8036	1.92	0.1817	1.645	0.4041	4.78	0.0118	0.73	0.9170	1.07	0.3949	3.115	0.4190	2.050	0.1939
Cd8b1	-1.355	0.0122	1.025	0.9924	-1.28	0.0585	-1.165	0.0250	5.665	0.1596	2.36	0.1293	-1.295	0.0291	0.34	0.7407	0.020	0.5189
Cd9	0.03	0.5370	1.105	0.5000	1.19	0.0172	0.015	0.5127	-1.655	0.0775	0.13	0.6081	1.045	0.4208	-1.14	0.0238	1.185	0.4440
Cd96	-1.225	0.0272	-1.035	0.0047	-1.785	0.0331	0.085	0.6596	7.985	0.4739	1.9	0.2721	-2.095	0.0462	0.04	0.5927	-2.415	0.0270
Cd97	1.875	0.1670	2.055	0.4617	1.905	0.0246	2.15	0.0880	3.78	0.2137	2.1	0.1257	2.43	0.1486	2.16	0.1404	2.580	0.1426
Cd99	0.145	0.5943	-1.195	0.0015	1.215	0.2892	0.165	0.6645	0.465	0.7928	-0.135	0.5630	0.485	0.7901	1.17	0.2487	0.105	0.6056
Cdh5	1.115	0.0826	0.075	0.6165	-1.205	0.0332	1.31	0.3648	1.92	0.1817	2.64	0.1413	-0.065	0.5202	1.29	0.1299	-0.110	0.5501
Cdkn1a	2.435	0.1291	2.02	0.2215	1.74	0.0515	2.795	0.2352	3.49	0.1956	0.31	0.7030	2.61	0.0119	3.03	0.0687	2.560	0.0041
Ceacam1	1.525	0.1939	1.055	0.3608	0.13	0.5981	1.315	0.2911	-0.28	0.5098	1.805	0.2840	1.655	0.2854	1.29	0.1087	1.845	0.0038
Cebpb	5.41	0.0790	3.715	0.3570	2.145	0.2918	4.135	0.0395	4.18	0.3560	4.185	0.3605	6.61	0.1178	4.52	0.0969	4.060	0.1821
Cfb	10.79	0.3781	5.02	0.4515	7.62	0.2944	15.82	0.0198	27.785	0.0030	5.39	0.2745	18.125	0.1285	5.065	0.2790	11.790	0.2864
Cfd	-4.325	0.1025	-4.815	0.0366	-5.71	0.3201	-3.135	0.0192	-4.395	0.0966	0.415	0.8103	-1.195	0.0333	-0.785	0.5973	-1.715	0.5603
Cfh	-1.455	0.0143	-1.315	0.0289	-1.015	0.0016	-1.725	0.0269	-3.03	0.2392	-0.025	0.5031	-1.785	0.0308	-1.695	0.0248	-1.240	0.0482
Cfi	-1.265	0.0070	1.265	0.2806	-1.45	0.0182	-0.075	0.5059	1.2	0.9455	2.03	0.3404	-0.175	0.5309	0.175	0.6203	-2.100	0.0308
Cfp	2.235	0.2515	2.525	0.3221	3.66	0.1020	2.75	0.0544	4.395	0.0570	3.24	0.3064	2.09	0.2786	3.265	0.1998	6.825	0.0060
Chuk	1.235	0.1967	-1.055	0.0108	-0.01	0.5000	1.26	0.0245	-0.3	0.5049	-1.285	0.0514	1.145	0.3039	0.025	0.5397	1.145	0.0656
Ciita	0.095	0.5687	2.45	0.4629	1.72	0.0441	2.16	0.1714	5.6	0.0662	3.245	0.4512	1.37	0.1997	1.82	0.1077	2.750	0.0218
Cish	1.43	0.2268	0.475	0.8044	-1.235	0.0441	-1.395	0.0754	19.115	0.4742	1.455	0.8856	1.295	0.0538	0.15	0.5974	-0.015	0.5031
Clec4a4	0.04	0.5781	1.4	0.4576	-1.53	0.0226	-1.615	0.0377	3.48	0.3895	0.09	0.5945	-1.595	0.0355	0.055	0.5496	-2.975	0.0360
Clec4e	13.71	0.3540	6.95	0.4392	2.68	0.0529	12.945	0.1973	14.31	0.2246	16.055	0.4309	22.79	0.0882	10.16	0.3564	8.520	0.2682
Clec5a	6.72	0.4047	3.96	0.3319	3.735	0.0244	10.885	0.2328	6.16	0.2892	4.3	0.4942	8.935	0.0897	4.285	0.3124	9.020	0.2046
Clu	0.095	0.5715	-1.075	0.0046	1.54	0.0821	1.375	0.2495	-1.92	0.0305	0.13	0.5930	1.175	0.1602	-1.26	0.0113	-0.160	0.5386
Cmklr1	1.865	0.1689	2.175	0.3712	1.35	0.2103	1.835	0.1168	3.48	0.1799	0.845	0.9504	1.945	0.0437	2.86	0.0648	2.885	0.0589
Cr2	-1.115	0.0075	-0.015	0.5123	-2.28	0.0175	-0.06	0.5367	2.545	0.4060	-0.215	0.5227	-2.26	0.0545	0.01	0.5949	-3.565	0.1206
Cradd	-1.4	0.0818	-1.23	0.0171	-1.54	0.0525	0.55	0.8362	0.185	0.6286	-2.84	0.1837	-1.415	0.0119	-1.31	0.0550	-1.865	0.0741
Crlf2	2.075	0.0266	1.72	0.2588	1.655	0.2193	1.695	0.1993	2.06	0.4970	0.545	0.8199	1.41	0.4219	1.765	0.0787	1.845	0.1082
Csf1	2.205	0.0918	1.725	0.2384	1.445	0.4016	2.645	0.0751	4.16	0.3204	-0.255	0.7123	1.99	0.0129	1.82	0.1669	1.445	0.0357
Csf1r	1.715	0.1941	2.055	0.2743	2.625	0.1143	2.445	0.1492	1.995	0.1477	2.065	0.3805	1.945	0.0437	2.155	0.1278	3.775	0.0218
Csf2	0.05	0.5604	0.035	0.5727	-1.715	0.0363	2.235	0.3145	7.005	0.3671	0.115	0.6090	-1.505	0.0495	0.15	0.6291	-1.650	0.1326
Csf2rb	2.075	0.0912	2.11	0.2789	1.625	0.1738	2.28	0.3800	5.53	0.2787	1.705	0.8383	2.59	0.1110	2.68	0.0416	1.765	0.0950
Csf3r	3.3	0.1957	2.39	0.3785	1.22	0.1145	3.825	0.1192	4.84	0.0033	0.91	0.9839	2.285	0.3001	2.91	0.0200	1.490	0.2578
Ctla4	-1.18	0.0379	2.28	0.4433	1.5	0.1500	0.25	0.6850	2.845	0.0190	2.91	0.0333	-0.095	0.5058	1.885	0.2966	1.255	0.3100
Ctnnb1	-1.065	0.0108	-1.16	0.0059	0.01	0.5217	-0.135	0.5110	-1.355	0.0660	0.24	0.6545	-0.095	0.5304	-1.36	0.0216	1.155	0.2869
Ctsc	2.96	0.2298	3.095	0.2596	3.385	0.1149	3.92	0.1743	3.445	0.2325	2.875	0.4966	3.33	0.0518	3.21	0.1498	7.025	0.1063
Ctsg	0.16	0.6233	-25.92	0.5029	-2.185	0.1520	39.49	0.5179	58.025	0.4924	1.395	0.9194	-1.99	0.0404	-1.32	0.0384	-2.585	0.0576
Ctss	6.295	0.3544	4.81	0.4184	10.99	0.2969	9.58	0.1338	5.48	0.2517	5.43	0.3336	7.59	0.0664	3.875	0.1717	12.685	0.0443
Cul9	-0.1	0.5624	-1.25	0.0028	-1.135	0.0164	-2.08	0.1714	1.365	0.4727	-2.275	0.1442	-1.625	0.0641	-1.435	0.0379	-2.255	0.0244
Cx3cl1	-0.08	0.5255	-1.16	0.0412	-0.055	0.5232	0.055	0.5380	0.675	0.8808	-1.355	0.0068	-1.32	0.0165	-1.16	0.0324	-0.150	0.5162
Cx3cr1	-1.415	0.0852	-1.02	0.0032	1.52	0.1900	-1.335	0.0014	1.71	0.3198	2.215	0.4241	-1.29	0.0083	-1.295	0.0429	-0.255	0.5978
Cxcl1	4.57	0.2653	1.78	0.4325	-1.385	0.0333	2.915	0.2783	4.14	0.2306	1.8	0.4752	3.205	0.0188	2.525	0.1176	-0.115	0.5685
Cxcl10	19.78	0.4492	4.88	0.4097	6.15	0.3349	54.055	0.1398	30.24	0.0374	4.08	0.2365	50.465	0.0740	5.265	0.3619	8.485	0.3220
Cxcl11	7.52	0.3581	3.875	0.4234	-1.08	0.0214	8.58	0.2816	11.22	0.0676	2.215	0.0549	6.725	0.1128	3.81	0.2197	2.335	0.3384
Cxcl12	1.43	0.1455	0.08	0.5537	1.555	0.2353	1.13	0.3855	0.04	0.6433	2.135	0.0028	1.465	0.2411	1.58	0.2952	2.365	0.0766
Cxcl13	2.125	0.1946	0.03	0.5399	-1.585	0.0283	0.27	0.6743	2.205	0.3563	-0.98	0.5111	1.26	0.4174	-0.09	0.5460	0.130	0.6105

Cxcl15	-1.085	0.0137	-0.115	0.5426	-1.88	0.1051	-0.065	0.5594	13.695	0.4704	9.97	0.5682	-1.49	0.0153	-1.295	0.0346	-5.220	0.1164
Cxcl3	14.05	0.3893	15.745	0.4090	2.26	0.1911	13.485	0.2018	27.94	0.0925	30.87	0.4510	39.89	0.0515	37.285	0.3816	13.915	0.4156
Cxcl9	22.87	0.4235	4.58	0.4136	11.255	0.3652	28.12	0.0413	20.22	0.3307	2.88	0.7197	24.635	0.0632	3.525	0.2477	10.545	0.2176
Cxcr1	-1.305	0.0317	0.085	0.5681	-1.67	0.0024	-0.15	0.5956	2.8	0.3414	-0.07	0.5174	-1.52	0.0554	-0.185	0.5027	-4.265	0.0645
Cxcr2	2.705	0.3473	2.11	0.4124	-0.395	0.5199	1.805	0.3215	3.925	0.2338	3.76	0.4930	1.585	0.1950	1.73	0.0952	-1.510	0.0329
Cxcr3	-1.245	0.0269	2.225	0.4894	1.77	0.0903	0.185	0.6446	3.79	0.1457	2.075	0.3397	-0.005	0.5063	2.13	0.3151	2.030	0.0981
Cxcr4	2.805	0.2787	2.54	0.2647	2.75	0.1872	4.085	0.2565	3.515	0.0442	3.59	0.3020	2.5	0.2710	2.86	0.0034	3.590	0.2129
Cxcr5	-1.24	0.0680	1.38	0.2393	-1.635	0.0947	-0.765	0.5482	3.96	0.3618	0.29	0.7136	-1.41	0.0684	-0.055	0.5525	-3.200	0.0694
Cxcr6	-1.15	0.0296	2.485	0.4778	2.91	0.1346	1.68	0.1821	4.98	0.4292	3.11	0.1395	-1.085	0.0046	1.885	0.3409	4.050	0.0167
Cybb	4.32	0.2654	3.555	0.3499	8.63	0.3670	9.01	0.0476	5.65	0.1479	3.525	0.4598	8.15	0.0736	3.205	0.2817	9.825	0.0191
Ddx58	3.52	0.2383	2.545	0.3312	2.43	0.2338	5.065	0.0956	3.68	0.0356	0.235	0.7287	4.46	0.0386	2.775	0.2499	2.775	0.2376
Defb1	-1.69	0.0870	-1.62	0.0988	-2.01	0.1232	-2.545	0.0296	-1.46	0.1152	-1.815	0.0620	-2.175	0.0351	-2.08	0.0103	-1.840	0.0045
Defb14	0	0.5032	-0.03	0.5295	-0.6	0.5230	0.02	0.5309	2.26	0.4208	3.84	0.0714	-1.63	0.0314	0.15	0.6336	-2.590	0.1729
Dpp4	-1.11	0.0121	0.185	0.6263	1.32	0.3834	-0.125	0.5767	1.585	0.1025	1.055	0.9834	0	0.5125	0.02	0.5338	0.050	0.5378
Ebi3	1.495	0.2163	1.275	0.2905	1.065	0.0489	0.55	0.8212	3.575	0.3566	1.775	0.0123	1.335	0.3632	1.755	0.0042	1.370	0.2452
Eef1g	1.035	0.0903	1.05	0.1257	0	0.5125	-1.175	0.0044	-1.075	0.0199	0.035	0.5401	-0.135	0.5394	-1.085	0.0198	1.045	0.2048
Emr1	2.53	0.2487	2.31	0.4242	2.475	0.0280	2.07	0.2066	4.015	0.1856	2.77	0.1104	2.185	0.0295	2.265	0.1695	3.860	0.0533
Entpd1	2.29	0.1361	2.05	0.3659	2.57	0.1889	1.94	0.0135	2.245	0.2453	0.86	0.9601	2.455	0.0371	2.235	0.2858	4.410	0.1625
Eomes	-0.045	0.5149	1.535	0.3475	-1.195	0.0391	-1.45	0.0415	2.81	0.2006	-0.16	0.5027	-1.245	0.0354	1.32	0.0987	-1.590	0.0147
Ets1	1.52	0.0610	2.435	0.4287	2.195	0.2768	1.86	0.0074	3.075	0.2721	1.845	0.2272	1.455	0.1310	2.285	0.1342	2.800	0.1051
Fadd	-1.35	0.0728	-1.24	0.0454	-1.58	0.0664	-1.225	0.0272	2.14	0.3171	0.17	0.6470	-1.535	0.0013	-1.365	0.0443	-2.220	0.0533
Fas	2.035	0.0886	1.635	0.2851	1.34	0.0930	2.055	0.4548	2.455	0.1849	3.1	0.3529	1.655	0.0340	1.5	0.2849	1.500	0.1619
Fasl	1.44	0.3907	0.975	0.9923	1.325	0.0294	1.575	0.1676	6.01	0.1315	2.405	0.2740	1.52	0.0731	1.7	0.1517	2.220	0.1136
Fcamr	-0.085	0.5087	0.05	0.5887	-0.45	0.5391	-0.205	0.5509	3.68	0.3513	1.78	0.2548	-0.225	0.5630	0.175	0.6476	-2.720	0.0359
Fcer1a	0.01	0.5095	-1.13	0.0179	-1.4	0.0844	0.045	0.5784	2.025	0.3486	1.565	0.2116	-1.825	0.0237	-1.125	0.0105	-2.295	0.0396
Fcer1g	6.48	0.3117	4.735	0.4334	6.995	0.2751	7.33	0.1847	8.67	0.0083	7.535	0.0365	9.15	0.0708	4.405	0.2281	10.895	0.0923
Fcgr1	11.66	0.3855	6.305	0.3977	5.59	0.1550	15.62	0.1167	12.745	0.2407	5.32	0.4933	17.63	0.1047	6.41	0.2785	12.270	0.1200
Fcgr2b	3.485	0.3295	3.525	0.3694	4.35	0.1311	4.26	0.1196	3.575	0.4007	4.51	0.3324	3.215	0.0502	3.04	0.2619	7.055	0.0759
Fcgr3	3.115	0.4056	3.42	0.3456	4.405	0.2082	3.315	0.0426	4.795	0.1459	3.14	0.6969	3.915	0.1365	2.92	0.2485	6.915	0.0425
Fcgr4	6.63	0.3780	5.625	0.4266	6.485	0.1531	8.765	0.1407	13.59	0.1170	6.96	0.3219	10.025	0.0307	3.755	0.2268	10.425	0.1067
Fcgrt	-1.475	0.0449	-1.24	0.0170	1.175	0.3440	-1.46	0.0696	-1.55	0.0225	-0.225	0.5671	-1.48	0.0487	-1.315	0.0261	0.345	0.7205
Fkbp5	-1.18	0.0204	0.02	0.5127	-1.355	0.0014	0.095	0.5603	1.485	0.4429	0.22	0.6402	-1.425	0.0459	1.1	0.3888	-1.230	0.0200
Fn1	-0.17	0.5133	0.26	0.6768	2.165	0.1933	-0.24	0.5000	2.13	0.3449	2.59	0.3603	0.02	0.6257	1.26	0.4018	1.375	0.4448
Folr4	-1.3	0.0028	-0.01	0.6537	-1.54	0.0872	-0.335	0.5877	3.37	0.4219	2.515	0.3729	-1.605	0.0110	-1.235	0.0384	-4.420	0.3017
Foxp3	0.05	0.5658	1.32	0.0792	-1.455	0.0220	-2.165	0.0432	4.785	0.3567	7.665	0.4406	-1.79	0.0410	1.24	0.1308	-2.410	0.0725
Frmpd4	-1.415	0.0119	-1.59	0.0393	-2.155	0.0071	-0.32	0.5628	3.26	0.4900	4.355	0.6840	-2.03	0.0607	-1.975	0.0459	-4.255	0.0562
Fyn	1.395	0.0563	0.66	0.8751	1.51	0.2270	1.25	0.1257	1.79	0.4135	2.8	0.3982	1.66	0.1514	1.655	0.0049	2.020	0.0622
G6pdx	1.05	0.1560	1.145	0.3375	-0.04	0.5061	1.175	0.2879	-0.755	0.5018	-1.425	0.0925	1.125	0.3440	1.105	0.5000	0.095	0.5742
Gapdh	-1.145	0.0104	-1.2	0.0087	-1.455	0.0427	-1.245	0.0298	-1.425	0.1053	-1.08	0.0153	-1.215	0.0043	-1.065	0.0170	-1.330	0.0027
Gata3	1.05	0.3440	0.13	0.6056	-1.495	0.1000	0.445	0.7695	2.68	0.3305	1.24	0.2048	-1.31	0.0413	1.455	0.3145	-2.070	0.0987
Gfi1	-1.34	0.0082	-0.02	0.5122	-1.9	0.0808	0.295	0.8055	5.095	0.4070	7.325	0.5929	-1.875	0.0409	0.14	0.6066	-2.960	0.0609
Gm10499	2.84	0.3169	1.975	0.4028	2.02	0.1530	3.65	0.1486	6.28	0.2686	4.87	0.2919	3.52	0.2470	2.41	0.0181	3.085	0.0472
Gp1bb	-0.32	0.5024	-1.27	0.0532	-1.405	0.0777	-0.215	0.5078	2.1	0.7910	-1.505	0.0921	-2.115	0.0072	-1.625	0.0351	-2.555	0.1452
Gpi1	-1.62	0.0606	-1.825	0.1322	-1.715	0.0851	-0.165	0.5456	1.03	0.9909	-1.505	0.0996	-1.71	0.0795	-1.82	0.1051	-2.730	0.1065
Gpr183	-1.205	0.0101	0.13	0.6105	-0.08	0.5058	0.785	0.9463	2.355	0.0258	0.095	0.6185	-0.015	0.5595	1.155	0.2171	-0.585	0.5817
Gpr44	-1.375	0.0121	-0.015	0.5031	-2.015	0.1422	1.22	0.4852	2.85	0.3286	3.18	0.4881	-1.825	0.0214	-1.135	0.0313	-3.550	0.0014
Gusb	1.575	0.1881	1.63	0.4208	3.315	0.1524	1.52	0.0610	1.72	0.1223	2.87	0.0204	1.615	0.2590	1.73	0.2630	3.055	0.1592
Gzma	16.4	0.3666	5.455	0.4622	7.53	0.3297	31.05	0.1851	37.825	0.0855	6.295	0.3277	15.99	0.2732	3.83	0.0112	28.140	0.4203
Gzmb	3.32	0.1404	5.03	0.4480	2.26	0.1198	4.09	0.0452	15.2	0.1534	2.435	0.4205	3.23	0.2540	3.83	0.0938	5.230	0.3124
H2-Aa	1.42	0.0454	2.325	0.4161	6.04	0.2870	2.055	0.1395	4.76	0.0524	2.925	0.2905	1.865	0.0477	1.525	0.0061	6.215	0.0963
H2-Ab1	1.585	0.1025	2.53	0.4260	6.685	0.3294	2.27	0.0847	4.655	0.1949	3.75	0.2402	2.42	0.0269	1.97	0.0328	6.040	0.0754
H2-DMa	1.4	0.5000	2.73	0.4007	2.185	0.1091	1.695	0.0684	3.25	0.3543	2.65	0.1838	1.33	0.2048	1.945	0.1552	3.680	0.1197
H2-DMb2	0.02	0.5158	1.535	0.3730	1.765	0.0540	-0.035	0.6064	4.135	0.3310	2.155	0.0795	-1.37	0.0907	0.325	0.7113	1.385	0.3608
H2-Ea-ps	0.195	0.7368	1.26	0.0245	-1.685	0.0036	-1.725	0.1144	7.96	0.4612	3.455	0.4070	-0.135	0.5028	-0.335	0.5360	-2.365	0.2003
H2-Eb1	1.11	0.3608	1.95	0.3955	4.685	0.2167	1.315	0.1295	2.775	0.2219	2.64	0.2858	1.55	0.1802	1.605	0.0368	4.170	0.0181
H2-K1	4.365	0.0444	2.345	0.3328	5.9	0.3045	5.055	0.0835	3.67	0.4093	2.475	0.2515	5.155	0.0451	2.985	0.2000	4.780	0.1646

H2-Ob	-1.45	0.0286	0.075	0.5477	-1.425	0.0249	0.33	0.7211	3.79	0.4375	0.12	0.5972	-1.42	0.0079	-0.115	0.5401	-1.595	0.0841
H2-Q10	0.15	0.6000	-0.06	0.5645	-1.62	0.0267	0.005	0.5304	2.295	0.0319	2.67	0.0114	-1.28	0.0084	0.1	0.5635	-1.995	0.0393
H60a	3.37	0.4905	0.1	0.6321	-2.68	0.1147	1.805	0.2375	13.435	0.5552	8.93	0.4559	1.51	0.4311	-0.115	0.5751	1.220	0.4003
Hamp	-1.44	0.0624	-0.015	0.5031	-1.785	0.0897	-1.44	0.0365	3.04	0.4706	1.855	0.8443	-1.465	0.0528	-1.505	0.0191	-3.135	0.0361
Hc	-0.57	0.5139	0.035	0.5727	-2.92	0.1452	-0.1	0.6428	9.31	0.4811	2.07	0.3116	-1.42	0.0785	-1.295	0.0732	-2.045	0.0386
Hcst	-0.05	0.5289	1.375	0.4028	-0.02	0.5031	1.445	0.8969	3.815	0.3196	0.185	0.7040	0.05	0.5959	1.65	0.1629	-1.120	0.0300
Hfe	-1.175	0.0219	-1.095	0.0167	1.165	0.0577	-0.09	0.5252	-1.445	0.0299	-0.02	0.5668	-1.265	0.0042	-1.23	0.0143	0.260	0.6788
Hif1a	1.245	0.2794	-0.03	0.5091	-1.105	0.0015	1.505	0.3076	-1.12	0.0270	-0.005	0.5915	1.365	0.4220	1.115	0.0826	-1.195	0.0159
Hlx	-1.33	0.0027	1.3	0.0212	-1.725	0.0269	1.04	0.9899	2.24	0.0614	-3.66	0.5140	-1.21	0.0173	1.175	0.3440	-1.640	0.0096
Hprt	-1.24	0.0057	-1.52	0.0829	-1.37	0.0536	-0.225	0.5152	-1.52	0.0804	0.34	0.7892	-0.08	0.5116	-1.425	0.0144	-1.805	0.0102
Icam1	2.815	0.2945	2.01	0.3858	1.8	0.0556	4.345	0.0786	4.005	0.0769	2.26	0.1392	3.215	0.1617	2.055	0.0392	2.125	0.1738
Icam2	1.125	0.2200	1.325	0.4112	1.28	0.2767	1.07	0.3305	5.28	0.4078	2.015	0.3822	1.21	0.0903	1.47	0.0941	1.725	0.1995
Icam4	-1.18	0.0058	-1.24	0.0651	-1.415	0.0981	-0.165	0.5287	0.95	0.9843	0.035	0.5623	-1.775	0.0241	-0.03	0.5637	-2.835	0.0025
Icam5	-1.655	0.0610	1.375	0.0927	-0.465	0.5906	-0.485	0.5247	5.99	0.4151	2.45	0.0829	-0.25	0.5051	1.395	0.0081	-2.610	0.0106
Icos	-0.05	0.5119	3.87	0.4314	3.11	0.0572	0.005	0.5156	9.99	0.0354	3.66	0.4120	0.3	0.6896	3.14	0.2039	3.865	0.2028
Icosl	1.18	0.2361	0.075	0.5591	-1.11	0.0151	1.255	0.1821	2.215	0.2095	0.105	0.6127	0.005	0.5275	1.19	0.2537	-0.300	0.5073
Ifi204	9.61	0.4159	6.76	0.4532	5.655	0.0890	12.92	0.0501	9.605	0.3938	5.385	0.3348	15.325	0.0202	5.44	0.2754	8.735	0.2244
Ifi35	2.765	0.2419	2.36	0.3315	2.065	0.1153	3.86	0.0356	3.48	0.1316	1.525	0.2370	4.555	0.0367	2.415	0.2797	2.525	0.2185
Ifih1	4.095	0.2310	2.86	0.3500	2.505	0.2212	6.05	0.0578	3.735	0.2947	2.315	0.0842	5.435	0.0394	3	0.2450	3.165	0.1924
Ifit2	10.865	0.3875	5.245	0.4079	2.81	0.2069	17.09	0.0008	18.305	0.1420	2.545	0.0391	21.9	0.1598	5.19	0.3864	6.875	0.2950
Ifitm1	1.53	0.1531	1.43	0.0737	1.805	0.2025	1.485	0.2088	0.125	0.6002	0.115	0.7244	1.535	0.0297	1.475	0.0734	2.320	0.0768
Ifna1	-1.195	0.0073	-1.14	0.0357	-1.645	0.0707	-1.33	0.0137	1.68	0.1112	0.085	0.5788	-2.63	0.0854	-0.005	0.6004	-5.525	0.0560
Ifna2	-0.025	0.5122	-1.155	0.0251	-1.8	0.0227	0.25	0.7194	4.09	0.2860	0.11	0.6061	-1.605	0.0110	-1.245	0.0241	-3.090	0.0621
Ifnar1	1.395	0.1951	1.39	0.2478	1.52	0.1788	1.51	0.0498	1.86	0.1455	1.365	0.3258	1.315	0.2228	1.66	0.0193	1.925	0.0309
Ifnar2	2.27	0.2033	2.165	0.3219	2.095	0.0377	3.28	0.1106	2.185	0.1349	-0.975	0.6741	2.26	0.1198	2.035	0.2213	2.860	0.1283
Ifnb1	1.8	0.0792	1.155	0.4788	-1.475	0.0424	1.64	0.3967	2.98	0.2361	0.02	0.6003	2.01	0.1245	2.405	0.1576	-3.140	0.1348
Ifng	-0.025	0.6072	1.385	0.4365	-0.055	0.5260	0.31	0.7085	3.72	0.3208	1.32	0.4344	-1.295	0.0457	1.545	0.0756	-0.240	0.5076
Ifngr1	1.65	0.0489	1.96	0.3312	2.215	0.1615	2.245	0.1089	0.28	0.7907	1.53	0.4878	1.98	0.0647	2	0.0445	3.550	0.0324
Ifngr2	1.5	0.1738	1.34	0.4496	1.31	0.1608	1.455	0.3949	0.35	0.7250	1.79	0.2024	1.33	0.1873	1.685	0.1505	1.645	0.0247
Igf2r	1.13	0.1900	0.27	0.6844	2	0.3393	1.595	0.3265	2.285	0.2962	2.28	0.3868	0.1	0.6003	1.475	0.1127	1.785	0.1396
Ikbkap	-1.165	0.0250	-0.015	0.5355	-1.305	0.0207	0.885	0.9684	1.07	0.4511	-2.205	0.2184	-1.315	0.0151	-1.075	0.0138	-1.600	0.0049
Ikbkb	1.24	0.0792	1.445	0.1339	1.305	0.4308	2.94	0.4170	1.79	0.2919	0.035	0.5727	1.195	0.2886	1.575	0.0277	1.520	0.0972
Ikbke	3.435	0.1501	2.985	0.3769	1.87	0.0292	3.585	0.0430	4.15	0.3056	3.655	0.2325	3.39	0.0637	3.7	0.0913	3.510	0.2326
Ikbkg	1.165	0.2389	-0.03	0.5151	-1.11	0.0241	0.735	0.9082	1.28	0.9251	-0.625	0.5172	1.165	0.1331	1.21	0.3072	-0.040	0.5030
Ikzf1	1.495	0.1695	2.31	0.4901	1.52	0.3772	1.2	0.2143	6.085	0.1266	1.735	0.3272	1.385	0.2852	1.77	0.1145	1.240	0.2284
Ikzf2	-0.07	0.5117	-1.17	0.0381	-1.315	0.0234	-1.225	0.0072	-1.73	0.1198	-1.575	0.0012	-1.555	0.0286	-1.435	0.0457	-1.535	0.0314
Ikzf3	0.02	0.5395	1.715	0.4817	-1.405	0.0040	-0.045	0.6055	3.23	0.0711	2.105	0.4369	-1.365	0.0013	1.51	0.2902	-1.175	0.0219
Ikzf4	1.15	0.3440	0.105	0.5776	-1.5	0.0886	1.915	0.2938	1.85	0.3440	-0.125	0.5111	-1.3	0.0166	-1.155	0.0369	-1.740	0.0023
Il10	-0.015	0.5212	1.595	0.1522	-1.56	0.1011	1.48	0.4263	5.185	0.3770	3.335	0.4422	0.12	0.5760	1.585	0.2432	-1.590	0.0270
Il10ra	6.635	0.3964	4.63	0.4270	4.555	0.1177	9.625	0.1948	13.33	0.1229	2.795	0.4892	10.525	0.0991	3.65	0.0384	6.985	0.1717
Il10rb	2.025	0.0031	1.845	0.2468	1.85	0.0819	1.965	0.1521	1.7	0.3024	2.07	0.0357	2.135	0.0809	2.085	0.1906	2.580	0.0242
Il11ra1	-0.355	0.5182	-1.32	0.0274	-0.145	0.5266	-1.195	0.0073	3.235	0.4623	5.82	0.4940	-1.215	0.0187	-1.295	0.0153	-2.185	0.0942
Il12a	0.1	0.6931	1.44	0.1284	-2.18	0.0777	-0.115	0.5376	12.36	0.4750	3.91	0.2801	1.94	0.1139	0.59	0.8440	-0.020	0.5092
Il12b	1.32	0.1371	1.34	0.2645	0.005	0.5473	1.41	0.3919	3.2	0.2928	1.69	0.4711	1.665	0.0430	1.51	0.1233	-1.585	0.1085
Il12rb1	1.365	0.1621	2.34	0.3426	0.025	0.5482	1.315	0.1488	4.93	0.1586	1.825	0.3497	1.16	0.2284	2.63	0.0312	2.070	0.3070
Il12rb2	3.935	0.3334	4.95	0.4364	3.065	0.1054	7.17	0.1759	28.895	0.1783	5.69	0.0555	2.545	0.2085	4.85	0.1695	8.200	0.2859
Il13	-1.375	0.0548	0.015	0.5663	-3.14	0.0476	-0.4	0.5282	4.145	0.3393	1.87	0.4612	-1.19	0.0232	-0.03	0.5031	-3.000	0.0540
Il13ra1	3.155	0.2705	2.425	0.3137	3.075	0.1749	4.07	0.1467	1.015	0.9964	2.34	0.3496	3.335	0.0746	2.7	0.2487	5.030	0.2324
Il15	4.47	0.0694	2.395	0.1018	1.335	0.4804	3.535	0.2183	4.435	0.2164	1.8	0.0318	4.55	0.0465	2.255	0.2169	1.955	0.2088
Il15ra	2.785	0.1181	1.695	0.3359	0.08	0.5861	1.885	0.1312	5.07	0.0794	1.615	0.2847	2.365	0.1620	2.74	0.1334	0.255	0.6655
Il16	1.155	0.1799	1.725	0.4866	1.135	0.2463	-1.28	0.0502	2.925	0.1320	1.935	0.3100	-1.175	0.0044	1.555	0.3199	1.420	0.2829
Il17a	-1.39	0.0900	0.065	0.5773	-2.27	0.0175	0.025	0.6051	3.965	0.4491	4.835	0.4303	-1.51	0.0304	0.055	0.5661	-4.125	0.1898
Il17b	-1.36	0.0538	1.315	0.1865	-2.645	0.2033	-0.28	0.5374	5.115	0.4446	0.31	0.7172	-1.415	0.0329	-1.285	0.0209	-3.325	0.1129
Il17f	-1.035	0.0078	1.12	0.2952	-1.535	0.0163	0.34	0.7105	1.295	0.1786	0.345	0.7307	-1.68	0.0190	0.065	0.5948	-2.675	0.0647
Il17ra	2.125	0.0927	2.015	0.3281	1.38	0.2098	1.745	0.0128	2.42	0.2075	2.595	0.1620	0.135	0.5934	2.075	0.0854	1.270	0.0471
Il17rb	-1.475	0.0603	1.37	0.3415	-0.645	0.5019	0.775	0.9537	7.49	0.4665	1.865	0.2727	0.865	0.9561	-0.13	0.5445	1.945	0.1615

Il17re	-1.72	0.0467	-1.39	0.0664	-1.84	0.0869	0.4	0.8179	1.625	0.4843	-1.57	0.0124	-1.56	0.0224	-1.18	0.0117	-2.685	0.0985
Il18	1.455	0.1836	1.13	0.2753	-1.115	0.0256	1.74	0.0172	1.445	0.2629	1.09	0.2048	1.355	0.1153	1.29	0.1916	1.105	0.0303
Il18r1	1.315	0.0101	1.775	0.3902	1.52	0.0972	1.675	0.3776	3.46	0.1051	1.99	0.2500	1.09	0.4208	1.715	0.2102	2.100	0.2118
Il18rap	0.015	0.5278	1.49	0.4585	-0.015	0.5695	0.2	0.6827	2.635	0.1435	-0.015	0.7659	-0.01	0.5184	1.435	0.3692	-1.135	0.0164
Il19	-1.215	0.0330	0.085	0.5653	-0.405	0.5155	-1.26	0.0113	2.16	0.0384	-0.255	0.5469	-1.98	0.0235	-0.19	0.5377	-5.385	0.1101
Il1a	3.535	0.2205	2.455	0.2127	1.33	0.3179	3.035	0.0016	5.765	0.1374	1.3	0.9301	5.735	0.0329	4.08	0.3131	3.905	0.4049
Il1b	8.515	0.3758	5.675	0.4291	2.365	0.2886	8.185	0.1600	11.605	0.0833	13.285	0.3677	13.01	0.2502	6.055	0.2360	4.215	0.2689
Il1r1	1.135	0.2048	1.145	0.3375	1.1	0.0635	-0.04	0.5149	1.335	0.1402	1.42	0.4511	1.245	0.2578	1.21	0.2829	-0.010	0.5819
Il1r2	3.1	0.2474	2.02	0.4603	1.385	0.0741	2.705	0.2432	3.155	0.0015	12.685	0.3973	2.395	0.2331	1.93	0.1543	1.730	0.2630
Il1rap	1.315	0.2405	1.175	0.2879	-1.12	0.0060	1.545	0.0756	1.375	0.2639	-0.875	0.5542	1.29	0.2308	1.08	0.2952	-1.115	0.0015
Il1rl1	1.445	0.2865	0.23	0.6946	0.045	0.5547	1.92	0.3007	2	0.1010	1.075	0.9774	-0.14	0.5190	-0.095	0.5000	0.040	0.5489
Il1rl2	1.73	0.2557	0.14	0.6187	1.325	0.3721	1.295	0.4412	2.765	0.2930	1.965	0.2805	1.61	0.3471	1.555	0.2452	2.600	0.0159
Il1rn	27.505	0.4111	9.62	0.4604	18.52	0.3236	49.595	0.0970	35.785	0.0512	15.73	0.3928	48.305	0.0017	13.32	0.3986	23.385	0.1954
Il2	1.09	0.3228	1.185	0.1840	-1.775	0.0172	-0.315	0.5386	2.55	0.1723	1.045	0.2048	-2.19	0.0676	-0.135	0.5394	-3.075	0.0336
Il20	-1.215	0.5884	1.765	0.3537	-3.845	0.0125	4.87	0.6611	26.61	0.4430	4.025	0.4936	1.495	0.0064	0.18	0.6461	1.530	0.2505
Il21	-1.2	0.0202	1.105	0.4332	-1.675	0.0416	-0.03	0.5662	3.68	0.3666	1.635	0.4089	-1.71	0.0539	0.035	0.6038	-2.280	0.0542
Il21r	1.65	0.2753	1.025	0.9925	1.94	0.3420	2	0.2849	8.95	0.2127	25.235	0.4728	1.485	0.1482	2.42	0.1872	1.355	0.1496
Il22	-1.63	0.0747	-1.255	0.0324	-1.95	0.0388	-0.125	0.6718	15.475	0.5420	0.675	0.8957	0.065	0.6262	-2.4	0.1184	-2.335	0.0410
Il22ra2	-0.905	0.5302	3.115	0.7122	-2.925	0.2087	1.27	0.9845	36.795	0.4622	1.03	0.9919	0.115	0.6706	0.135	0.5960	1.200	0.4097
Il23a	-0.125	0.5111	0.025	0.5454	-1.735	0.0430	0.005	0.6685	2.135	0.2985	0.115	0.5838	-2.43	0.0922	-0.06	0.5287	-3.965	0.0860
Il23r	-1.225	0.0528	-1.16	0.0088	-1.88	0.0155	0.155	0.6180	2.605	0.0139	-1.385	0.0862	-2.115	0.0540	0.035	0.5596	-4.200	0.1934
Il25	-1.325	0.0014	-1.195	0.0304	-1.68	0.1013	-1.135	0.0253	1.61	0.2653	1.2	0.3888	-2.015	0.0684	-1.105	0.0317	-3.715	0.0128
Il27	1.265	0.4039	0.165	0.6142	-1.55	0.0013	-1.095	0.0046	4.94	0.3803	3.685	0.3412	1.25	0.3624	1.535	0.1904	-2.175	0.0331
Il27ra	-1.15	0.0266	2.11	0.4971	1.12	0.3743	0.525	0.8135	4.255	0.0537	0.555	0.8765	0.025	0.5221	0.445	0.7856	1.290	0.4401
Il28a	-1.58	0.0271	-0.105	0.5085	-2.105	0.0886	0	0.5778	2.115	0.3867	-0.105	0.5168	-1.69	0.0402	0	0.5274	-4.400	0.0283
Il2ra	6.735	0.3046	4.405	0.4110	5.05	0.3840	21.375	0.3988	67.995	0.3811	2.115	0.7899	6.625	0.0255	4.615	0.1628	12.945	0.4153
Il2rb	2.44	0.1434	3.41	0.4637	2.255	0.0932	2.855	0.2161	5.935	0.3766	2.35	0.4678	2.44	0.1094	2.97	0.0387	4.305	0.1985
Il2rg	3.56	0.1513	3.825	0.4516	2.885	0.1315	4.055	0.1621	8.935	0.0739	2.965	0.1782	3.21	0.2237	3.22	0.1079	3.930	0.0864
Il3	-1.39	0.0584	0.115	0.5942	-2.72	0.2425	0.34	0.7604	4.075	0.4462	1.22	0.1423	-1.16	0.0236	0.155	0.6056	-1.235	0.0384
Il33	2.15	0.0607	1.59	0.0538	1.335	0.3065	1.87	0.1369	1.355	0.0626	1.31	0.0205	1.515	0.2304	1.32	0.1745	1.430	0.2268
Il4	-1.31	0.0248	1.095	0.0997	-1.88	0.0813	0.35	0.7365	3.04	0.3829	-0.1	0.6184	-1.725	0.0222	-0.015	0.5792	-2.300	0.0116
Il4ra	3.065	0.2604	4.45	0.3454	3.665	0.0983	4.945	0.1603	5.46	0.1472	3.255	0.4579	3.295	0.1511	4.75	0.0777	5.815	0.0951
Il5	1.19	0.1947	2.065	0.0861	-2.835	0.1012	1.805	0.8539	8.745	0.4557	-0.035	0.6125	1.705	0.4717	-0.085	0.5584	1.485	0.4654
Il6	4.935	0.1965	1.995	0.2677	-1.485	0.0803	4.33	0.1519	2.18	0.2552	-2.945	0.5637	5.275	0.0097	3.425	0.2872	-0.820	0.5484
Il6ra	1.735	0.2355	0.63	0.8620	1.405	0.4008	2.01	0.1838	3.285	0.1117	2.155	0.2531	1.57	0.0668	1.455	0.0628	1.315	0.2746
Il6st	1.345	0.0826	1.15	0.4028	1.29	0.2498	1.38	0.5000	-0.195	0.5445	1.325	0.0683	1.495	0.4935	1.215	0.2892	1.450	0.1120
Il7	-0.595	0.5137	-0.06	0.5522	-1.865	0.1661	-1.31	0.0138	4.64	0.3983	1.91	0.1040	-1.41	0.0013	0.04	0.6173	-1.855	0.0766
Il7r	3.55	0.2182	3.375	0.3765	2.54	0.0984	4.2	0.2353	5.12	0.2518	5.035	0.4822	3.83	0.1287	5.01	0.1192	4.395	0.0234
Il9	-1.67	0.0072	1.105	0.4332	-2.145	0.0354	-1.66	0.1481	1.935	0.4283	0.29	0.7136	-1.79	0.0951	0.145	0.7026	-3.825	0.0729
Ilf3	-1.115	0.0135	-1.16	0.0412	-1.33	0.0572	-1.315	0.0644	0.28	0.6759	-2.115	0.1083	-1.16	0.0177	-1.135	0.0104	-2.000	0.0613
Irak1	1.18	0.1392	1.265	0.4750	-1.125	0.0105	-1.16	0.0118	1.505	0.3366	-0.88	0.5133	-0.17	0.5185	1.085	0.0374	-0.060	0.5446
Irak2	1.645	0.1501	1.41	0.1813	1.155	0.3193	1.84	0.0378	2.255	0.2214	-0.07	0.5953	1.71	0.1325	1.68	0.0281	1.190	0.5000
Irak3	1.845	0.0564	1.67	0.3065	1.01	0.5000	1.195	0.2886	2.34	0.0237	2.665	0.1301	1.325	0.1257	1.795	0.0757	1.260	0.3512
Irak4	1.515	0.1280	1.75	0.1894	1.365	0.0435	1.595	0.0267	1.405	0.4839	1.735	0.3272	1.175	0.2578	1.71	0.0803	1.745	0.1058
Irf1	3.335	0.2518	2.97	0.2887	3.68	0.2601	5.565	0.0063	4.74	0.3283	3	0.3322	5.39	0.0751	2.865	0.1426	4.340	0.2952
Irf3	-1.37	0.0081	-1.04	0.0125	-1.735	0.0845	-0.445	0.5885	2.045	0.4140	-1.725	0.0408	-1.58	0.0468	0.05	0.5788	-2.360	0.0623
Irf4	-1.335	0.0177	0.045	0.5629	-1.535	0.0213	-1.36	0.0054	2.11	0.2501	-0.805	0.5070	-1.77	0.0138	1.185	0.3783	-2.265	0.1053
Irf5	3.97	0.2591	3.19	0.3921	2.24	0.0665	4.73	0.1443	7.365	0.0255	2.37	0.4391	4.92	0.0760	3.32	0.1351	4.080	0.0822
Irf7	5.19	0.2909	3.36	0.3956	2.735	0.2856	8.32	0.1034	8.415	0.1496	2.71	0.2569	13.13	0.1464	3.725	0.2167	4.290	0.2328
Irf8	2.945	0.2609	2.6	0.4271	2.145	0.1342	4.1	0.1799	4.715	0.2810	3.005	0.1146	2.05	0.0665	2.205	0.0710	2.995	0.2303
Irgm1	4.265	0.3449	3.545	0.3723	6.25	0.3396	6.485	0.0319	12.16	0.0450	3.67	0.2807	8.36	0.0303	2.535	0.1289	3.745	0.2006
Itga2b	-1.545	0.0013	0.05	0.5838	-1.65	0.1094	-1.53	0.0949	2.97	0.4514	1.955	0.3961	-2.06	0.0021	-1.255	0.0324	-3.410	0.1351
Itga4	1.96	0.3697	2.435	0.4887	1.905	0.1418	1.725	0.1425	12.025	0.3829	4.505	0.3974	1.895	0.0249	2.065	0.0388	2.605	0.1119
Itga5	3.185	0.1025	2.305	0.3589	1.685	0.2430	3.055	0.0847	5.355	0.1705	3.435	0.0922	2.08	0.1834	2.765	0.1640	1.980	0.1093
Itga6	1.205	0.0465	0.09	0.5844	1.225	0.3440	0.035	0.5430	-1.305	0.0592	-0.215	0.5483	1.215	0.5000	1.18	0.0353	1.075	0.2780
Itgal	2.67	0.0834	3.245	0.4734	3.185	0.1556	2.91	0.0796	11.475	0.0185	2.87	0.3100	2.595	0.0140	3.33	0.1241	4.120	0.2012

Itgam	2.66	0.0574	2.215	0.3540	2.715	0.0684	2.545	0.0718	3.495	0.0497	2.93	0.3017	2.765	0.0198	3.075	0.1286	4.375	0.1912
Itgax	0.145	0.6021	1.805	0.4796	1.785	0.0041	1.205	0.1669	4.57	0.1755	2.81	0.0421	-1.08	0.0214	1.65	0.4235	1.625	0.3440
Itgb1	1.4	0.3201	1.4	0.4576	1.785	0.1550	1.485	0.1482	1.57	0.0223	1.715	0.1444	1.3	0.0424	1.335	0.2439	2.165	0.0464
Itgb2	3.15	0.2137	3.92	0.4234	3.025	0.0016	3.29	0.1342	6.29	0.0096	3.37	0.4447	3.09	0.0183	3.51	0.0228	4.260	0.1083
Itln1	-1.21	0.0173	-1.43	0.0314	-1.515	0.0467	-0.22	0.5569	2.765	0.1774	1.515	0.2827	-2.13	0.0487	-0.28	0.5074	-4.355	0.0681
Jak1	1.26	0.0972	1.305	0.2476	1.295	0.0323	1.1	0.2422	1.81	0.2983	1.43	0.3127	1.105	0.0903	1.285	0.0112	1.460	0.1096
Jak2	2.695	0.0131	1.775	0.3193	1.375	0.0255	3.115	0.1117	2.755	0.0847	0.18	0.6566	2.945	0.0115	2.42	0.1411	2.100	0.2619
Jak3	1.57	0.2442	1.815	0.3752	1.275	0.3927	2.24	0.2079	3.06	0.0247	1.755	0.3811	1.27	0.2048	1.925	0.1840	1.560	0.1125
Kir3dl1	0.075	0.6395	0.77	0.9282	-1.52	0.0604	8.51	0.6007	41.005	0.4919	3.75	0.3232	1.57	0.0778	0.275	0.7074	3.165	0.2815
Kir3dl2	-1.15	0.0118	-1.125	0.0344	-1.505	0.0343	-0.11	0.5352	1.8	0.2422	-0.01	0.5520	-2.44	0.0737	-0.095	0.5507	-4.075	0.0532
Kit	-1.78	0.1678	-1.6	0.1091	-1.86	0.0267	0.09	0.7440	2.565	0.7775	0.7	0.9023	-1.95	0.0667	-1.595	0.0355	-2.220	0.1135
Klra1	-0.11	0.5113	-0.075	0.5145	-1.79	0.0727	0.09	0.5792	2.765	0.4453	0.665	0.8930	-2.655	0.0200	0.02	0.6003	-4.160	0.0049
Klra21	0.06	0.6831	2.165	0.3384	-1.72	0.6123	0.665	0.9597	22.005	0.4510	4.865	0.0681	2.81	0.4125	2.08	0.1834	1.660	0.8541
Klra4	-0.055	0.6280	0.42	0.7884	-3.215	0.0128	0.055	0.5740	13.075	0.3649	1.755	0.8657	0.465	0.7852	1.115	0.4711	-1.355	0.0633
Klra5	0.49	0.8959	1.675	0.1437	-2.14	0.0827	-0.05	0.5651	16.405	0.4603	3.455	0.4070	1.035	0.9902	1.475	0.2364	3.035	0.7095
Klra6	-1.195	0.0044	0.355	0.7243	-1.555	0.0658	0.21	0.6690	4.155	0.2073	1.83	0.3043	-1.335	0.0746	0.18	0.6607	-0.050	0.5060
Klra7	1.845	0.3647	2.615	0.4580	-1.415	0.0774	1.72	0.2736	6.105	0.0886	3.295	0.4540	-1.525	0.0290	0.335	0.7168	-1.185	0.0538
Klra8	0.01	0.5306	1.61	0.4433	-0.275	0.5098	1.265	0.4481	3.01	0.1159	-0.735	0.5612	-2.605	0.0256	0.04	0.6109	-1.595	0.0331
Klrb1	-0.38	0.5244	0.135	0.6786	-2.565	0.0259	9.975	0.4972	59.285	0.4781	2.91	0.0894	0.485	0.7976	1.06	0.1051	-2.890	0.1213
Klrc1	1.21	0.4845	2.4	0.4268	1.645	0.2649	-0.07	0.5543	3.28	0.4043	3.84	0.0336	-1.145	0.0015	2.155	0.3072	2.620	0.0118
Klrc2	0.29	0.8086	2.38	0.4583	-1.95	0.0752	-1.725	0.1480	7.695	0.4044	1.58	0.2683	1.7	0.2805	1.47	0.2897	1.935	0.3410
Klrc3	-0.075	0.7551	1.615	0.2931	-2.135	0.2103	2.26	0.4393	12.25	0.4487	1.56	0.9049	0.37	0.7942	0.25	0.7240	0.730	0.9504
Klrd1	2.21	0.3097	3.18	0.4514	2.29	0.1123	2.875	0.3390	6.225	0.2542	5.405	0.1483	-0.13	0.5445	1.72	0.2662	2.875	0.0458
Klrk1	12.145	0.4292	4.715	0.4299	5.1	0.1581	28.07	0.3142	25.035	0.2482	3.805	0.3117	23.685	0.1868	3.395	0.2370	13.280	0.3364
Lair1	2.1	0.3483	2.385	0.4287	2.225	0.0903	2.515	0.1702	6.61	0.2069	2.405	0.4186	1.74	0.0344	1.83	0.1064	3.500	0.0483
Lck	1.83	0.2859	3.505	0.4479	3.43	0.2025	2.55	0.3001	6.45	0.3651	2.765	0.2419	3.135	0.1049	3.64	0.1004	5.540	0.1853
Lcp2	-0.82	0.6235	0.56	0.8864	-4.12	0.1962	-1.71	0.1093	3.435	0.2883	1.92	0.4785	0.055	0.5524	1.19	0.3586	-1.160	0.0412
Lef1	-0.01	0.5093	1.315	0.0101	0.085	0.6817	1.75	0.4398	3	0.3127	1.355	0.4414	1.1	0.1257	1.47	0.1204	0.040	0.5432
Lif	2.495	0.2521	2.015	0.1567	-1.225	0.0329	1.525	0.0903	3.07	0.1245	1.965	0.1333	1.455	0.0210	2.385	0.0069	-1.165	0.0191
Lilra5	-1.305	0.0455	0.025	0.5310	-2.21	0.1022	-1.195	0.0333	1.555	0.3541	-1.75	0.1190	-1.755	0.0289	-1.515	0.0392	-2.275	0.0185
Lilra6	0.255	0.6739	1.305	0.0727	-1.74	0.0441	0.025	0.5915	10.14	0.4565	1.845	0.4806	0.075	0.5647	1.43	0.1313	-1.905	0.0449
Lilrb3	10.32	0.3831	6.71	0.4476	7.79	0.3191	9.59	0.0407	14.895	0.0768	4.69	0.4272	9.625	0.1704	4.915	0.2379	10.735	0.0062
Lilrb4	11.665	0.3649	6.09	0.4340	6.1	0.2226	16.53	0.0209	8.36	0.2108	6.985	0.3285	11.22	0.0676	5.115	0.2434	12.175	0.1872
Litaf	3.245	0.0915	2.545	0.2622	3.06	0.0921	3.76	0.0666	3.235	0.0327	1.435	0.8935	3.115	0.0406	3.125	0.1199	3.570	0.0740
Lta	-1.25	0.0255	0.05	0.5711	-1.685	0.1366	-0.035	0.5559	3.11	0.3954	1.27	0.4208	-1.44	0.0856	-0.01	0.5242	-1.950	0.0108
Ltb	1.635	0.0251	3.1	0.4087	2.62	0.1870	2.925	0.2798	4.24	0.3716	1.27	0.9304	2.205	0.1428	2.745	0.2125	3.375	0.1049
Ltb4r1	1.31	0.2351	1.635	0.4152	-1.69	0.1101	0.045	0.5734	3.22	0.1328	2.275	0.1775	0	0.5704	1.555	0.3373	-1.435	0.0845
Ltb4r2	-1.335	0.0368	0.1	0.5852	-1.98	0.1509	0.01	0.6325	2.86	0.4086	2.715	0.2794	-1.355	0.0041	-0.27	0.5099	-3.875	0.0372
Ltbr	1.735	0.0988	1.475	0.2594	1.41	0.0310	1.92	0.1689	2.295	0.2619	1.31	0.4320	1.365	0.0781	1.66	0.0768	1.700	0.0724
Ltf	-1.285	0.0790	0.16	0.6137	-2.135	0.1096	-1.085	0.0198	2.81	0.4508	-1.275	0.0683	-1.605	0.0549	0.235	0.6748	-2.900	0.0940
Ly86	2.685	0.3800	2.285	0.3563	3.61	0.1577	3.255	0.1563	2.78	0.1063	2.975	0.1863	2.9	0.0833	1.745	0.3300	3.760	0.0984
Ly96	2.575	0.2879	2.08	0.3449	1.875	0.1872	3.52	0.1149	4.13	0.2661	2.535	0.0435	2.305	0.1228	1.945	0.2288	3.115	0.1577
Maf	1.305	0.1136	1.435	0.2683	1.115	0.0826	1.345	0.1538	1.8	0.2353	1.125	0.0255	0	0.5245	1.655	0.1294	1.195	0.3855
Map4k1	2.53	0.2270	2.83	0.4912	1.985	0.0355	2.12	0.1980	17.2	0.2939	4.9	0.0618	3.185	0.4200	2.665	0.1555	3.475	0.0347
Map4k2	-0.21	0.5052	-0.02	0.5489	-1.52	0.0680	1.285	0.1845	2.18	0.4348	-1.625	0.0206	-1.505	0.0621	-1.16	0.0441	-1.540	0.0175
Map4k4	1.485	0.0589	1.33	0.3875	1.675	0.1257	1.6	0.1459	1.73	0.3763	-0.175	0.6496	1.665	0.0430	1.77	0.1924	1.810	0.0704
Mapk1	0.015	0.5365	0.035	0.5430	1.08	0.2284	1.085	0.3100	-1.97	0.1854	-1.485	0.1078	-1.255	0.0521	0.005	0.5156	1.310	0.1217
Mapk11	-1.385	0.0200	-0.11	0.5113	-1.605	0.0281	-0.255	0.5124	2.415	0.4020	0.1	0.5954	-1.735	0.0151	-1.245	0.0354	-2.810	0.0200
Mapk14	0.035	0.5542	-0.02	0.5515	-1.105	0.0257	-0.065	0.5366	0.01	0.5095	0.365	0.7281	-0.055	0.5060	-1.09	0.0091	-1.060	0.0062
Mapkapk2	1.63	0.2228	1.265	0.1303	1.35	0.2578	1.57	0.1742	1.46	0.3679	0.08	0.6147	1.515	0.3495	1.65	0.0294	1.495	0.0831
Marco	-1.1	0.5476	2.465	0.2191	-1.68	0.1013	0.22	0.7706	12.805	0.3816	2.31	0.4120	1.31	0.3790	1.33	0.2389	2.990	0.7177
Masp1	1.18	0.3228	0.045	0.5547	0.075	0.5591	1.175	0.9545	12.415	0.4718	2.1	0.7880	-1.125	0.0165	1.37	0.5000	-0.170	0.5699
Masp2	-0.365	0.5092	-0.315	0.5571	-1.645	0.1340	36.845	0.5239	57.52	0.4981	1.165	0.3608	-1.535	0.0514	-0.525	0.5481	-12.760	0.0347
Mbl2	-1.38	0.0107	1.105	0.3949	-2.125	0.1040	0.27	0.7305	2.595	0.3375	1.855	0.4728	-1.645	0.0180	-1.225	0.0186	-4.820	0.0371
Mbp	-1.775	0.0674	-1.58	0.0761	-1.245	0.0099	-1.425	0.0171	-2.285	0.0781	-1.285	0.0403	-1.05	0.0031	-1.82	0.0203	-0.430	0.5621
Mif	1.02	0.2952	0.045	0.5346	-0.14	0.5163	1.09	0.2048	-0.25	0.5337	1.265	0.4618	1.05	0.1257	1.145	0.1916	-1.225	0.0129

Mme	-1.38	0.0214	-1.145	0.0163	-1.49	0.0662	-2.015	0.1301	-1.205	0.0216	-0.01	0.5623	-1.43	0.0183	-1.395	0.0252	-1.645	0.0205
Mr1	-1.315	0.0289	-1.37	0.0161	-1.3	0.0111	-1.555	0.0037	-1.19	0.0493	-1.36	0.0081	-1.4	0.0371	-1.605	0.0086	-0.190	0.5305
Ms4a1	-1.28	0.0279	-0.055	0.5369	-1.345	0.0823	-0.23	0.5543	2.625	0.4235	1.165	0.2048	-1.71	0.0586	-0.075	0.5173	-0.340	0.5714
Msr1	6.5	0.3927	4.275	0.4349	3.9	0.0916	6.66	0.0259	10.39	0.0722	6.35	0.0380	5.94	0.1234	4.17	0.1798	6.565	0.1149
Muc1	-1.02	0.0063	-0.04	0.5236	-0.195	0.5422	-1.29	0.0692	-1.645	0.0325	1.115	0.4052	1.115	0.2375	-1.245	0.0014	-0.010	0.5357
Mx1	0.26	0.6768	0.05	0.6051	-2.085	0.0811	1.54	0.1051	0.295	0.7722	-0.195	0.5053	1.22	0.1423	1.48	0.1051	-3.140	0.0826
Myd88	2.525	0.2859	2.58	0.4186	1.38	0.3820	2.75	0.0145	2.74	0.3510	2.45	0.1466	2.015	0.0219	2.58	0.0040	1.905	0.1148
Ncam1	-1.44	0.0052	-1.45	0.0389	-1.405	0.0198	-1.45	0.0286	-2.92	0.1514	-0.34	0.5337	-1.59	0.0319	-1.83	0.0561	-2.790	0.0736
Ncf4	7.425	0.3337	5.18	0.4552	4.43	0.1228	9.185	0.2088	8.4	0.1816	6.585	0.1461	10.275	0.1851	4.645	0.1922	8.560	0.1279
Nfatc1	1.74	0.4913	0.655	0.8899	2.065	0.3846	0.05	0.5550	16.68	0.4044	6.47	0.4080	2.19	0.1215	1.7	0.0992	2.950	0.0456
Nfatc2	-0.045	0.5206	1.235	0.4567	-0.025	0.5210	0.715	0.9035	1.64	0.2457	-1.025	0.6041	-1.48	0.0538	1.4	0.4665	1.110	0.5000
Nfatc3	1.13	0.1900	-0.035	0.5237	1.155	0.3193	1.285	0.3171	1.815	0.4310	0.01	0.5306	1.065	0.0489	1.1	0.3440	0.045	0.5434
Nfil3	1.945	0.0034	1.52	0.1674	0.09	0.5656	2.69	0.1372	2.67	0.2330	1.77	0.4742	1.875	0.1116	2.17	0.0865	1.575	0.3832
Nfkb1	1.715	0.0665	0.43	0.7702	1.395	0.3762	2	0.0445	3.33	0.2627	1.265	0.4192	1.265	0.2191	1.435	0.1087	1.420	0.1631
Nfkb2	2.785	0.1420	1.695	0.4560	1.515	0.0677	2.21	0.0263	3.475	0.0526	2.54	0.0413	2.66	0.0421	2.48	0.1023	1.960	0.0066
Nfkbia	3.295	0.0787	1.79	0.3111	1.38	0.2952	2.57	0.0926	2.505	0.2397	1.87	0.3990	3.765	0.1825	2.79	0.1568	1.990	0.1207
Nfkbiz	2.12	0.1506	1.595	0.2207	1.305	0.4784	2.44	0.0965	0.725	0.8999	0.325	0.7284	2.08	0.1166	2.09	0.2392	1.710	0.1994
Nod2	5.48	0.3042	4.56	0.4363	1.645	0.4408	5.62	0.3147	28.005	0.3341	5.5	0.0311	6.68	0.0202	3.66	0.1683	4.320	0.2393
Nos2	5.305	0.0714	2.525	0.1456	-1.23	0.0257	2.26	0.1725	7.945	0.0050	4.55	0.3142	2.12	0.2720	4.05	0.1884	2.465	0.4223
Notch1	1.065	0.1444	0.25	0.6647	1.04	0.4097	0.135	0.5881	1.635	0.1805	1.355	0.2313	1.125	0.0760	1.435	0.1916	-1.135	0.0045
Notch2	1.235	0.4717	0.05	0.5658	1.21	0.2578	1.26	0.4174	1.46	0.0138	0.04	0.5756	1.265	0.0599	1.39	0.1288	1.225	0.4208
Nox1	-1.285	0.0431	-1.195	0.0159	-1.545	0.0287	-1.41	0.0684	1.825	0.3770	-0.13	0.5165	-2.345	0.0935	-1.44	0.0753	-4.415	0.0417
Nox3	-0.16	0.5000	-1.125	0.0284	-2.115	0.0702	-0.145	0.5880	2.55	0.2243	1.88	0.4535	-1.96	0.0792	-0.11	0.5274	-4.850	0.0780
Nox4	1.305	0.4179	1.265	0.1976	1.49	0.0519	-0.005	0.6310	0.75	0.9097	1.215	0.3977	1.52	0.1560	1.39	0.4745	1.995	0.3141
Npc1	1.165	0.2716	1.2	0.0635	1.05	0.1257	1.295	0.3247	-0.015	0.5744	-0.26	0.5635	-0.125	0.5111	1.125	0.2200	1.175	0.1257
Nt5e	-1.845	0.0768	-1.735	0.1072	-1.375	0.0335	-1.97	0.0726	-1.59	0.1167	-1.54	0.0550	-2.03	0.0753	-1.865	0.0233	-1.680	0.0780
Oaz1	-1.11	0.0151	-1.2	0.0520	-1.215	0.0101	-0.165	0.5107	-2.115	0.1103	-1.37	0.0376	-1.14	0.0416	-1.375	0.0013	-1.265	0.0042
Pax5	-1.54	0.0200	0.045	0.5629	-1.445	0.0039	-1.81	0.0945	2.79	0.3610	-0.16	0.5027	-1.735	0.0291	-1.36	0.0645	-2.945	0.0523
Pdcd1	1.1	0.1855	1.875	0.4735	1.665	0.1183	0.135	0.6205	3.33	0.1293	2.025	0.3836	1.52	0.2338	2.225	0.1257	1.250	0.2422
Pdcd1lg2	-0.035	0.5031	0.06	0.6038	-1.495	0.1173	-0.23	0.5388	2.295	0.1519	0.04	0.5517	-1.565	0.0778	0.02	0.5666	-2.515	0.0962
Pdcd2	-1.12	0.0240	1.105	0.3529	-1.325	0.0451	-1.465	0.0142	4.76	0.4745	-1.125	0.0045	-0.125	0.5165	-0.025	0.5487	-1.565	0.0037
Pdgfb	-0.165	0.5000	0.22	0.6581	0.04	0.5255	2.29	0.4848	9.535	0.4760	-0.155	0.6278	-1.54	0.0500	1.3	0.4892	-1.410	0.0866
Pdgfrb	-1.44	0.0287	-1.145	0.0163	-1.26	0.0028	0.22	0.6494	1.575	0.3275	-0.49	0.5400	-1.43	0.0026	-0.06	0.5547	-2.095	0.1070
Pecam1	1.595	0.0903	1.63	0.4015	1.905	0.2501	1.635	0.2256	0.29	0.7690	2.005	0.0032	1.34	0.0746	1.765	0.1973	2.500	0.1855
Phlpp1	-1.22	0.0600	-1.185	0.0451	-1.145	0.0104	0.035	0.5514	-1.125	0.0195	-2.33	0.1555	-1.705	0.0129	-1.255	0.0549	-1.555	0.0087
Phlpp2	1.335	0.3356	-1.035	0.0016	-1.25	0.0283	1.71	0.0979	1.425	0.8934	-2.45	0.1625	-1.375	0.0495	-1.24	0.0426	-2.345	0.0257
Pigr	1.24	0.3362	1.05	0.4296	-1.41	0.0132	-1.16	0.0177	1.595	0.1215	0.405	0.7701	-1.605	0.0378	-0.14	0.5190	-1.620	0.0582
Pla2g2a	-1.295	0.0153	-1.495	0.0674	-1.445	0.0713	1.805	0.8518	2.13	0.3769	-2.19	0.1470	-2.295	0.0855	0.23	0.6596	-3.925	0.1454
Pla2g2e	-1.2	0.0548	-1.235	0.0100	-1.77	0.0069	1.265	0.4481	1.835	0.5000	-1.775	0.1541	-1.895	0.0253	-1.03	0.0031	-2.545	0.0063
Plau	2.34	0.3356	2.565	0.3748	1.99	0.1145	2.375	0.1781	2.955	0.0244	4.27	0.3579	2.31	0.1200	2.715	0.0903	2.935	0.0639
Plaur	3.545	0.3306	2.715	0.3849	1.64	0.1928	5.64	0.1714	4.795	0.1664	4.655	0.3208	4.745	0.0331	3.745	0.0955	2.790	0.1126
Pml	2.375	0.1478	2.025	0.2814	1.485	0.0459	3.19	0.0174	2.435	0.3355	2.655	0.3066	2.645	0.1242	2.405	0.1099	1.820	0.2164
Polr1b	-1.26	0.0310	-1.06	0.0124	-1.48	0.0333	0.05	0.5378	2.96	0.4117	1.99	0.8139	-1.41	0.0632	-0.115	0.5028	-2.415	0.0382
Polr2a	0.045	0.5734	-1.205	0.0043	-1.11	0.0181	-0.09	0.5509	0.335	0.7114	-1.36	0.0243	-1.635	0.0278	-1.2	0.0462	-1.555	0.0162
Pou2f2	2.09	0.0640	1.865	0.4849	1.15	0.1659	1.715	0.2338	2.71	0.1913	0.23	0.6910	1.505	0.0315	1.88	0.0720	1.225	0.1257
Pparg	-0.02	0.5031	-0.075	0.5116	-0.19	0.5603	-1.24	0.0397	2.41	0.1335	2.125	0.4765	-1.58	0.0468	-1.145	0.0163	-2.115	0.0601
Ppbp	-1.435	0.1100	2.15	0.3400	1.62	0.8914	0.12	0.6119	2.755	0.2556	7.67	0.4971	1.355	0.1325	1.615	0.3988	1.740	0.0770
Ppia	0.01	0.5217	1.15	0.3440	1.145	0.0219	-0.145	0.5189	-1.44	0.0572	-0.38	0.5157	0.03	0.5672	-1.045	0.0109	1.375	0.1257
Prdm1	-0.13	0.5321	1.33	0.1331	-0.005	0.5726	1.85	0.0225	2.505	0.4324	0.165	0.6828	-1.24	0.0397	1.355	0.0803	0.180	0.6257
Prf1	3.01	0.3721	4.185	0.4540	1.9	0.1984	3.03	0.0188	12.97	0.0835	4.42	0.1568	2.065	0.0090	3.745	0.1943	4.615	0.2834
Prim1	1.17	0.1112	1.11	0.3179	-1.235	0.0214	-1.075	0.0015	1.37	0.0686	1.16	0.5000	-1.315	0.0590	-1.045	0.0016	-1.230	0.0456
Prkcd	2.37	0.0510	1.7	0.4615	2.015	0.1026	2.585	0.1742	2.49	0.1224	1.7	0.4126	2.5	0.1093	2.215	0.1858	2.540	0.0781
Psmb10	3.205	0.3693	2.76	0.4472	3.485	0.2833	4.435	0.0306	11.645	0.3034	0.725	0.9188	7.89	0.2622	2.91	0.2028	3.105	0.0588
Psmb11	-1.59	0.0927	-0.235	0.5387	-2.37	0.1644	-1.62	0.1200	5.835	0.3897	3.305	0.4510	-1.715	0.0828	-0.245	0.5222	-3.260	0.0448
Psmb5	1.125	0.2200	1.155	0.4320	-0.005	0.5154	-0.005	0.5094	-1.03	0.5108	1.2	0.2422	0.16	0.6036	0.08	0.5704	1.185	0.3541
Psmb7	1.175	0.2879	0.005	0.5246	-1.065	0.0015	0.055	0.5409	-3.485	0.3100	0.2	0.6281	1.15	0.0424	-0.015	0.5355	1.280	0.2383

Psmb9	3.285	0.3747	2.525	0.3992	1.91	0.1964	3.695	0.2062	7.675	0.0942	2.965	0.0598	8.16	0.3796	2.74	0.1087	2.905	0.1587
Psmc2	1.28	0.0454	1.225	0.1526	1.14	0.1344	1.14	0.3949	-0.035	0.5150	1.26	0.2548	1.16	0.3262	0.08	0.5537	1.345	0.2688
Psmd7	1.23	0.2375	1.11	0.4365	0.095	0.5715	1.15	0.3119	-1.315	0.0726	-0.95	0.5032	1.22	0.1423	1.28	0.2767	1.325	0.0489
Ptafr	5.915	0.3143	4.005	0.4310	2.525	0.0230	4.59	0.1002	8.475	0.1974	3.735	0.3632	6.305	0.1157	4.12	0.2373	3.780	0.0820
Ptger4	-0.09	0.5143	0.285	0.7057	0.12	0.5895	-1.27	0.0196	4.19	0.2616	1.84	0.0604	-1.145	0.0163	1.4	0.2284	-0.040	0.5911
Ptgs2	8.36	0.2762	6.82	0.4100	3.795	0.0011	7.945	0.0362	6.195	0.1613	5.075	0.1317	9.96	0.1364	6.475	0.3033	9.755	0.3273
Ptk2	1.065	0.2338	-1.26	0.0506	-1.32	0.0137	1.05	0.1257	-1.375	0.0442	-0.065	0.5470	0.02	0.5338	-1.13	0.0090	-1.540	0.0251
Ptpn2	1.56	0.0454	0.07	0.5879	1.14	0.1344	1.64	0.0987	0.065	0.6177	-0.215	0.5438	1.3	0.0424	1.175	0.0182	1.315	0.4897
Ptpn22	1.775	0.1018	2.42	0.4719	1.905	0.0035	1.665	0.4428	4.575	0.0365	2.195	0.2222	1.57	0.0056	2.515	0.1224	2.070	0.1234
Ptpn6	4.25	0.2355	3.5	0.4185	3.82	0.2750	4.515	0.0425	5.82	0.2298	3.65	0.0694	4.58	0.0142	3.425	0.1028	6.015	0.1149
Ptprc	8.36	0.3519	4.87	0.4608	7.33	0.3083	10.535	0.0490	12.21	0.1670	4.98	0.3178	13.21	0.0809	4.22	0.1935	9.975	0.1680
Rae1	1.225	0.2299	0.005	0.5418	-1.085	0.0107	0.005	0.5304	-0.01	0.5865	1.15	0.0844	-1.275	0.0266	1.065	0.0489	-1.120	0.0150
Rag1	-0.04	0.5236	1.165	0.3608	-2.02	0.0755	0.03	0.6226	2.74	0.4155	4.995	0.4837	-1.56	0.0012	-0.095	0.5627	-3.605	0.0269
Rag2	-1.08	0.0061	1.105	0.0303	-2.28	0.0677	-2.545	0.1507	2.625	0.2704	1.875	0.4574	-1.76	0.0231	-1.49	0.0789	-4.095	0.0789
Rela	1.89	0.1610	1.405	0.2048	1.305	0.1535	1.945	0.1488	3.525	0.3569	1.6	0.3594	1.805	0.2511	1.635	0.1712	2.070	0.1291
Relb	2.42	0.0847	1.8	0.4045	1.375	0.1894	2.285	0.2679	2.79	0.2311	0.435	0.7807	2.6	0.0159	2.425	0.0379	1.885	0.0610
Rorc	-1.32	0.0710	-1.76	0.0023	-1.83	0.0472	-0.31	0.5279	0.05	0.6226	-1.555	0.0386	-1.56	0.0050	-1.48	0.0282	-2.105	0.0562
Rpl19	1.14	0.2184	1.14	0.1772	1.195	0.1750	0.055	0.5438	-0.095	0.5278	1.13	0.3512	1.265	0.4039	1.135	0.3228	1.475	0.0734
Runx1	1.575	0.0717	1.745	0.1945	2.015	0.1269	1.605	0.0785	2.525	0.0686	1.92	0.1625	-1.08	0.0092	1.66	0.1145	1.800	0.1484
Runx3	1.295	0.0538	2.25	0.4137	1.275	0.0116	1.93	0.8086	6.88	0.2587	0.86	0.9626	-1.255	0.0042	2.45	0.4677	1.275	0.0347
S100a8	10.72	0.2809	6.85	0.4329	3.94	0.2126	10.52	0.1080	9.985	0.1036	56.9	0.4888	11.915	0.0550	9.16	0.2639	9.645	0.3241
S100a9	11.505	0.2345	10.04	0.4576	5.985	0.1925	28.96	0.1157	20.945	0.0714	67.6	0.4877	17.845	0.0179	16.445	0.3783	16.300	0.3749
Sdha	-1.315	0.0316	-1.315	0.0590	-1.27	0.0643	-1.325	0.0342	-2.155	0.0473	-1.385	0.0227	-1.335	0.0095	-1.43	0.0026	-1.225	0.0386
Sele	1.575	0.2375	-1.22	0.0057	-1.71	0.0562	0.07	0.6000	2.59	0.3156	1.39	0.1900	-0.055	0.5260	0.045	0.5784	-3.485	0.0163
Sell	7.785	0.3751	3.885	0.4291	1.48	0.2167	9.94	0.1003	7.89	0.3237	4.235	0.1791	9.065	0.2563	4.035	0.2111	2.435	0.3152
Selplg	0	0.5303	-0.19	0.5948	-1.675	0.0511	-0.15	0.5508	5.33	0.3665	0.355	0.7260	-1.56	0.0075	-0.125	0.5767	-3.355	0.0907
Serping1	2.38	0.2007	1.91	0.4388	2.175	0.2492	2.675	0.1110	2.335	0.3793	1.755	0.0463	2.24	0.2261	2.035	0.0765	2.835	0.0913
Sh2d1a	-3	0.0870	-0.04	0.5347	-1.585	0.0332	-2.73	0.2419	1.655	0.4181	-1.815	0.0170	-2.28	0.1394	-1.96	0.1680	-1.230	0.0228
Sigirr	-1.43	0.0988	-0.085	0.5487	-1.38	0.0214	0.175	0.6678	2.92	0.3604	1.305	0.0313	-1.46	0.0155	-0.065	0.5312	-1.925	0.0597
Ski	-1.02	0.0016	1.15	0.4296	-0.07	0.5284	-0.115	0.5593	1.8	0.2692	0.065	0.5799	-1.1	0.0182	1.33	0.2220	-1.270	0.0698
Slamf1	-1.095	0.0167	1.525	0.2578	-0.13	0.5295	0.47	0.7927	3.9	0.1734	3.54	0.3824	1.23	0.1363	1.79	0.0402	1.130	0.4471
Slamf7	20.945	0.3859	7.335	0.4444	8.355	0.2213	39.105	0.0843	21.025	0.0411	5.055	0.3420	29	0.0425	7.335	0.2787	15.325	0.1883
Smad3	1.255	0.1112	0.035	0.5430	-0.025	0.5092	1.265	0.1303	2.49	0.3936	1.86	0.4690	1.1	0.2422	1.14	0.2578	-1.110	0.0211
Smad5	1.045	0.3228	-1.265	0.0126	-0.065	0.5088	1.1	0.1855	-1.32	0.0055	0.1	0.6404	-1.15	0.0237	-1.325	0.0041	-1.380	0.0374
Socs1	4.7	0.1696	3.05	0.3348	2.18	0.1885	8.395	0.0919	11.015	0.1644	4.75	0.2018	6.975	0.0208	3.8	0.2344	4.085	0.2406
Socs3	3.585	0.2654	4.31	0.3994	3.165	0.3518	5.7	0.1113	8.115	0.0753	4.29	0.0636	4.465	0.0668	4.985	0.2547	3.150	0.1594
Spn	1.36	0.1051	1.715	0.8453	1.4	0.3078	1.37	0.4124	8.295	0.1552	0.995	0.9988	1.685	0.0046	2.565	0.1225	2.575	0.0982
Src	1.21	0.4845	-1.2	0.0318	-0.16	0.5000	1.42	0.3743	-0.05	0.5148	-1.1	0.5030	-1.305	0.0207	-0.08	0.5172	-1.355	0.0338
Stat1	2.995	0.2219	3.19	0.4310	4.185	0.2493	4.905	0.0204	5.615	0.3091	2.345	0.4234	4.48	0.0201	2.57	0.0122	3.560	0.3224
Stat2	4.11	0.1233	3.395	0.4484	4.365	0.0350	11.715	0.2135	7.29	0.1033	3.615	0.3609	6.55	0.0046	2.405	0.0113	1.765	0.4459
Stat3	1.865	0.0404	1.735	0.3713	1.965	0.0755	2.72	0.0222	1.75	0.3502	1.475	0.3634	1.96	0.2898	1.97	0.0719	2.020	0.0991
Stat4	-0.065	0.5935	2.08	0.4818	1.385	0.3949	0.12	0.5997	6.24	0.1502	1.77	0.4742	0.225	0.6772	1.825	0.0193	1.525	0.2879
Stat5a	1.29	0.1916	1.185	0.3783	0.035	0.5623	0.185	0.6333	2.15	0.1150	-0.15	0.6299	1.09	0.4626	1.43	0.3127	-1.130	0.0209
Stat5b	-1.045	0.0109	1.065	0.1444	-0.015	0.5153	-0.085	0.5087	1.19	0.4254	-0.2	0.5205	-1.175	0.0102	1.08	0.2284	-1.100	0.0182
Stat6	1.65	0.1810	1.465	0.4562	1.465	0.0342	1.665	0.0620	5.25	0.4305	2.09	0.1764	1.795	0.0200	1.675	0.1166	2.125	0.0424
Syk	2.335	0.0877	2.32	0.3608	1.705	0.1204	2.235	0.3531	3.18	0.1183	2.47	0.2504	2.185	0.0722	2.67	0.1020	2.470	0.0903
Tagap	3.305	0.2891	1.865	0.4309	1.275	0.1257	2.745	0.1278	9.935	0.3331	2.465	0.0757	2.74	0.0873	2.48	0.0515	1.575	0.1573
Tal1	-1.3	0.0497	1.03	0.3743	-1.28	0.0195	0.285	0.6850	0.56	0.8281	-0.245	0.5076	-1.94	0.0583	-0.1	0.5196	-2.625	0.0255
Tap1	4.235	0.2525	3.685	0.3833	3.84	0.1686	5.85	0.0964	8.385	0.1139	2.775	0.4680	5.9	0.0078	3.005	0.0016	3.745	0.2555
Tapbp	3.255	0.1240	2.525	0.3379	2.665	0.2773	4.525	0.0460	1.65	0.8606	0.905	0.9725	4.38	0.0620	2.7	0.1400	3.470	0.2133
Tbk1	1.575	0.2564	1.395	0.4661	1.28	0.0454	1.58	0.0980	2.44	0.2128	1.43	0.1594	1.835	0.0946	1.65	0.2833	1.590	0.1381
Tbp	-1.045	0.0109	1.075	0.2048	-0.06	0.5089	1.39	0.2753	0.085	0.5708	-1.975	0.1801	0.09	0.5792	0.04	0.5315	1.400	0.3322
Tbx21	-0.05	0.5577	0.295	0.7593	-1.985	0.1253	1.535	0.4753	12.795	0.4497	3.82	0.3041	-1.465	0.0039	1.21	0.5000	-43.150	0.4538
Tcf4	-1.05	0.0155	-1.125	0.0045	1.045	0.3228	-1.115	0.0135	0.155	0.6779	0.285	0.6985	-1.17	0.0117	-1.155	0.0192	-1.175	0.0161
Tcf7	-0.16	0.5000	0.48	0.8045	1.275	0.3440	0.085	0.5625	2.985	0.0908	1.525	0.4615	-1.355	0.0122	0.235	0.6560	-0.435	0.5066
Tfrc	-1.355	0.0284	-1.61	0.0753	-1.835	0.0101	-0.09	0.5252	-2.695	0.1850	-1.38	0.1008	-1.645	0.0132	-1.4	0.0080	-1.695	0.1088

Tgfb1	2.745	0.0091	2.215	0.3761	2.265	0.1315	3.17	0.1160	3.62	0.0073	0.9	0.9705	2.965	0.0437	3.125	0.1054	4.785	0.1398
Tgfb2	-1.08	0.0031	-1.245	0.0128	-0.025	0.5092	0.005	0.5125	-1.735	0.0198	-1.41	0.0053	-1.43	0.0288	-1.46	0.0516	-1.635	0.0638
Tgfb3	-1.76	0.0369	-0.42	0.5045	-1.125	0.0135	0	0.6647	-2.135	0.0577	-2.77	0.1977	-1.85	0.0246	-1.715	0.0293	-1.935	0.1603
Tgfbi	2.765	0.1907	2.375	0.3641	2.22	0.1535	3.315	0.0807	3.875	0.1861	1.805	0.4203	3.295	0.0512	3.105	0.1865	3.605	0.1383
Tgfbr1	1.05	0.4296	0.02	0.5309	1.185	0.3020	-1.12	0.0330	1.05	0.5000	1.415	0.4843	-1.175	0.0424	0.015	0.5127	1.210	0.4682
Tgfbr2	1.075	0.1257	0.23	0.6462	1.635	0.3245	-1.11	0.0091	1.73	0.3055	1.3	0.3743	1.275	0.1257	1.32	0.0397	1.780	0.2266
Thy1	-0.015	0.5212	1.37	0.4731	1.345	0.1006	-1.205	0.0159	1.53	0.0718	0.015	0.5714	-0.165	0.5237	1.305	0.2993	1.830	0.1505
Tigit	-0.45	0.5129	1.665	0.5000	0.025	0.5454	1.895	0.4334	11.74	0.3865	2.89	0.4897	1.045	0.3228	1.49	0.4280	1.195	0.1131
Tirap	1.255	0.0622	0.015	0.5188	-1.455	0.0990	0.37	0.7557	1.865	0.1757	2.125	0.4243	0.045	0.5376	1.305	0.0313	-1.270	0.0364
Tlr1	2.685	0.1140	2.725	0.3521	1.95	0.0201	2.18	0.1534	3.87	0.0111	2.245	0.2838	2.175	0.1152	2.285	0.0470	3.010	0.0974
Tlr2	2.825	0.2874	2.175	0.4178	1.53	0.0240	3.59	0.1952	8.09	0.2934	0.79	0.9564	3.965	0.0589	2.855	0.2490	2.025	0.0650
Tlr3	1.89	0.3204	1.545	0.3248	-0.26	0.5289	2.285	0.1247	2.41	0.0941	1.235	0.1718	1.94	0.1781	1.67	0.0850	-1.270	0.0224
Tlr4	1.765	0.2123	1.8	0.2422	1.645	0.0345	1.545	0.0175	0.415	0.7907	1.67	0.4702	1.94	0.1905	1.775	0.1646	2.560	0.0041
Tlr5	-1.145	0.0341	-1.08	0.0153	-1.67	0.1155	0.055	0.5740	2.88	0.4161	1.605	0.4428	0.105	0.5856	-0.225	0.5102	-2.365	0.0519
Tlr8	2.45	0.2231	3.45	0.3285	2.12	0.1719	2.47	0.0173	5.055	0.0509	2.76	0.4852	2.28	0.1035	2.64	0.1189	4.640	0.0714
Tlr9	3.65	0.1509	3.085	0.4080	1.87	0.0656	4.445	0.1756	3.725	0.1742	0.465	0.8356	3.895	0.2225	3.12	0.1418	1.870	0.2244
Tmem173	2.41	0.2824	2.515	0.3816	1.575	0.0277	2.325	0.2607	3.31	0.1974	3.105	0.3423	2.79	0.0673	2.91	0.0499	2.870	0.1727
Tnf	2.51	0.0630	2.01	0.2977	0.065	0.5694	3.11	0.1017	3.685	0.1411	0.185	0.7959	3.165	0.1843	3.565	0.1685	1.715	0.2102
Tnfaip3	-0.085	0.5143	-1.515	0.0843	-1.735	0.0151	-1.11	0.0211	0.05	0.5318	-1.49	0.1138	-1.17	0.0439	-0.05	0.5344	-1.920	0.0909
Tnfaip6	1.4	0.1409	1.265	0.2402	0.295	0.7227	1.235	0.0135	1.475	0.5000	1.36	0.0704	1.87	0.3942	0.205	0.6287	1.700	0.3827
Tnfrsf11a	0.04	0.5489	-0.015	0.5355	-1.845	0.0525	-0.005	0.5124	2.065	0.0861	1.21	0.0303	-1.675	0.1049	-0.06	0.5259	-3.160	0.0245
Tnfrsf13b	0.245	0.7057	1.635	0.4515	-1.43	0.0497	-0.325	0.5702	4.925	0.3295	5.59	0.2979	0.26	0.6727	1.655	0.0630	1.135	0.3904
Tnfrsf13c	-0.225	0.5250	0.49	0.9181	-2.94	0.1025	4.26	0.4921	27.92	0.4816	1.26	0.9589	-0.055	0.6850	-0.66	0.5429	-17.805	0.3408
Tnfrsf14	2.21	0.0052	2.215	0.4492	2.485	0.1775	2.33	0.0096	3.215	0.2567	2.7	0.2550	3.185	0.1801	2.43	0.0267	3.485	0.1374
Tnfrsf17	-1.38	0.0187	1.115	0.5000	-1.645	0.0492	-1.14	0.0386	3.83	0.4803	0.44	0.8505	-1.32	0.0247	-1.255	0.0014	-3.455	0.1602
Tnfrsf1b	3.235	0.1603	3.305	0.4212	3.585	0.1061	3.145	0.0015	3.56	0.3224	4.19	0.0990	4.865	0.1758	4.315	0.1075	4.995	0.0422
Tnfrsf4	-0.055	0.5288	1.43	0.2892	0.12	0.6448	0.035	0.5851	2.35	0.0330	-0.015	0.7307	-1.095	0.0258	1.61	0.2017	0.030	0.5484
Tnfrsf8	1.805	0.3949	0.36	0.7417	1.04	0.0903	1.94	0.0271	6.055	0.4872	3.2	0.4822	1.15	0.2422	1.17	0.4386	0.155	0.6131
Tnfrsf9	0.61	0.8539	0.055	0.5467	-2.105	0.0481	0.605	0.8606	10.29	0.4592	3.91	0.2801	1.665	0.0048	1.645	0.3709	-1.300	0.0111
Tnfsf10	1.935	0.3751	2.025	0.4674	1.475	0.1886	2.42	0.0759	5.565	0.2409	1.355	0.2313	2.07	0.3519	1.345	0.2533	1.595	0.3265
Tnfsf11	1.2	0.3888	0.59	0.8403	0.405	0.7994	1.575	0.3907	9.995	0.4273	3.36	0.7032	1.265	0.2607	2.14	0.0167	1.635	0.0351
Tnfsf12	-1.335	0.0123	-1.145	0.0311	-1.46	0.0491	-1.27	0.0392	-1.745	0.0636	-1.76	0.0092	-2.68	0.0138	-1.995	0.0730	-2.095	0.0031
Tnfsf13b	1.075	0.3440	-0.03	0.5295	-1.385	0.0678	0.055	0.6809	3.28	0.4488	1.555	0.2550	-1.055	0.0139	1.39	0.4745	1.040	0.4097
Tnfsf14	1.305	0.4784	1.955	0.2776	0.01	0.6381	2.555	0.4014	2.505	0.0653	-1.195	0.6618	-1.29	0.0665	1.54	0.3228	-1.295	0.0125
Tnfsf15	-0.28	0.5193	1.175	0.0544	-1.7	0.0283	0.015	0.5505	4.385	0.3831	1.35	0.4405	-1.57	0.0396	0.07	0.5588	-2.275	0.0126
Tnfsf18	-1.215	0.0302	-1.045	0.0016	-1.66	0.0974	1.25	0.3973	2.1	0.1641	1.17	0.4603	-2.215	0.0148	-0.195	0.5445	-4.800	0.1956
Tnfsf8	-0.255	0.5124	1.355	0.2916	-0.41	0.5401	-1.51	0.0932	6.11	0.4638	3.355	0.4363	-0.04	0.5061	1.09	0.0704	-1.320	0.0137
Tollip	-0.03	0.5295	-0.095	0.5278	-1.17	0.0293	-1.205	0.0159	-1.185	0.0015	-1.81	0.1252	-1.125	0.0195	1.035	0.3949	-1.355	0.0176
Traf1	3.765	0.1349	2.9	0.4757	2.69	0.1336	6.94	0.0873	11.56	0.2810	2.555	0.3009	5.79	0.1600	3.1	0.1402	2.385	0.1115
Traf2	1.28	0.0227	1.275	0.3267	-1.1	0.0212	1.495	0.3228	2.38	0.3205	2.625	0.4378	1.34	0.2487	1.265	0.2607	0.050	0.5577
Traf3	1.155	0.4788	0.22	0.6425	1.1	0.2952	0.315	0.7216	1.395	0.1195	-0.075	0.5363	-1.51	0.0279	1.155	0.0205	-1.325	0.0014
Traf4	-1.63	0.0290	-0.08	0.5282	-1.7	0.0588	-0.145	0.5028	1.86	0.4120	2.115	0.4824	-1.55	0.0225	-0.035	0.5237	-2.765	0.0245
Traf5	-0.015	0.5270	0.01	0.5157	-1.08	0.0122	1.23	0.3275	1.375	0.2495	-2.095	0.1601	-1.655	0.0012	-1.095	0.0288	-1.110	0.0271
Traf6	1.09	0.0704	1.075	0.0424	1.405	0.0704	1.76	0.0997	1.45	0.0704	0.35	0.7412	-0.03	0.5734	1.285	0.2631	1.555	0.2929
Trem1	2.37	0.2062	1.945	0.4291	-1.385	0.0387	1.77	0.3608	5.395	0.2567	3.17	0.4494	1.505	0.3640	1.455	0.0210	-0.480	0.5188
Trem2	-0.065	0.5545	2.315	0.3095	3.555	0.1049	1.045	0.3228	2.9	0.4064	5.045	0.2580	-1.15	0.0207	1.535	0.1795	3.730	0.3759
Trp53	1.25	0.0255	1.31	0.3349	-0.08	0.5282	2.5	0.4594	2.275	0.2401	-1.56	0.5134	-1.07	0.0031	1.865	0.2416	-1.150	0.0296
Tslp	-1.215	0.0101	0.105	0.6032	-1.215	0.0530	1.425	0.4924	2.905	0.3914	1.42	0.4332	-0.2	0.5420	1.27	0.2463	-1.890	0.0484
Tubb5	1.23	0.1096	1.39	0.3512	1.39	0.0326	1.17	0.1112	2.305	0.2764	1.25	0.3053	1.525	0.0544	1.495	0.1575	1.580	0.0656
Tyk2	0.02	0.5219	-0.14	0.5343	-1.195	0.0044	1.4	0.2558	2.23	0.2931	-0.195	0.5328	-1.455	0.0530	0.125	0.6124	-1.590	0.0368
Tyrobp	4.795	0.3275	4.035	0.3431	6.58	0.1809	5.53	0.0589	3.19	0.3683	3.88	0.4711	5.3	0.0296	3.24	0.1693	9.420	0.0468
Ube2l3	0.005	0.5125	-0.02	0.5462	1.025	0.5000	-1.075	0.0077	-1.2	0.0116	-1.35	0.0054	-1.16	0.0059	-1.025	0.0016	1.100	0.2952
Vcam1	3.645	0.2721	2.81	0.4057	2.74	0.0908	6.535	0.0874	2.985	0.4170	2.095	0.0838	5.725	0.0824	3.135	0.2425	3.790	0.2171
Vtn	-1.67	0.0500	-1.98	0.0933	-2.365	0.0274	-1.885	0.0649	-0.965	0.5219	-2.13	0.1166	-1.985	0.0245	-3.22	0.0617	-4.830	0.0131
Xbp1	1.345	0.0644	1.21	0.1772	1.435	0.0365	1.51	0.1233	0.3	0.6855	0.235	0.6603	1.39	0.1131	1.61	0.4046	2.125	0.3228
Xcl1	1.86	0.4073	1.83	0.2982	-0.13	0.5111	1.885	0.1852	3.665	0.0060	0.6	0.8561	0.03	0.5370	1.22	0.0863	0.000	0.5215

Xcr1	-1.17	0.0235	-1.24	0.0142	-1.34	0.0865	-3.455	0.2597	5.545	0.4790	1.335	0.0285	-2.1	0.0328	-0.235	0.5341	-2.655	0.2043
Zap70	0.03	0.5399	0.55	0.8371	1.15	0.0844	0.705	0.8952	5.96	0.2785	1.905	0.3653	1.18	0.1051	1.59	0.3488	1.340	0.2160
Zbtb7b	1.17	0.2160	1.075	0.4028	-1.28	0.0014	1.18	0.3228	4.56	0.4826	1.305	0.0313	0.06	0.5411	1.445	0.0783	-1.280	0.0335
Zeb1	-1.015	0.0016	-1.13	0.0060	-1.085	0.0229	-1.325	0.0151	-1.505	0.1168	-1.285	0.0125	-1.25	0.0536	-1.125	0.0075	-1.330	0.0027
